# Cross-sectional and longitudinal associations between active commuting and patterns of movement behaviour during discretionary time: A compositional data analysis

**DOI:** 10.1371/journal.pone.0216650

**Published:** 2019-08-16

**Authors:** Louise Foley, Dorothea Dumuid, Andrew J. Atkin, Katrien Wijndaele, David Ogilvie, Timothy Olds

**Affiliations:** 1 MRC Epidemiology Unit & UKCRC Centre for Diet and Activity Research (CEDAR), School of Clinical Medicine, University of Cambridge, Cambridge, England, United Kingdom; 2 School of Health Sciences, University of South Australia, Adelaide, South Australia, Australia; 3 School of Health Sciences, Faculty of Medicine and Health Sciences, University of East Anglia, Norwich, England, United Kingdom; 4 MRC Epidemiology Unit, School of Clinical Medicine, University of Cambridge, Cambridge, England, United Kingdom; Macquarie University Faculty of Medicine and Health Sciences, AUSTRALIA

## Abstract

**Background:**

Active living approaches seek to promote physical activity and reduce sedentary time across different domains, including through active travel. However, there is little information on how movement behaviours in different domains relate to each other. We used compositional data analysis to explore associations between active commuting and patterns of movement behaviour during discretionary time.

**Methods and findings:**

We analysed cross-sectional and longitudinal data from the UK Biobank study. At baseline (2006–2010) and follow up (2009–2013) participants reported their mode of travel to work, dichotomised as active (walking, cycling or public transport) or inactive (car). Participants also reported activities performed during discretionary time, categorised as (i) screen time; (ii) walking for pleasure; and (iii) sport and do-it-yourself (DIY) activities, summed to produce a total. We applied compositional data analysis to test for associations between active commuting and the composition and total amount of discretionary time, using linear regression models adjusted for covariates. Adverse events were not investigated in this observational analysis. The survey response rate was 5.5%. In the cross-sectional analysis (n = 182,406; mean age = 52 years; 51% female), active commuters engaged in relatively less screen time than those who used inactive modes (coefficient -0.12, 95% confidence interval [CI] -0.13 to -0.11), equating to approximately 60 minutes less screen time per week. Similarly, in the longitudinal analysis (n = 4,323; mean age = 51 years; 49% female) there were relative reductions in screen time in those who used active modes at both time points compared with those who used inactive modes at both time points (coefficient -0.15, 95% confidence interval [CI] -0.24 to -0.06), equating to a difference between these commute groups of approximately 30 minutes per week at follow up. However, as exposures and outcomes were measured concurrently, reverse causation is possible.

**Conclusions:**

Active commuting was associated with a more favourable pattern of movement behaviour during discretionary time. Active commuters accumulated 30–60 minutes less screen time per week than those using inactive modes. Though modest, this could have a cumulative effect on health over time.

## Introduction

Movement behaviours (i.e. physical activity and sedentary behaviour) are associated with health in adults.[1–3] Analyses simulating the effects of substituting behaviours with each other suggest that replacing sedentary time with physical activity lowers the risk of chronic disease and mortality.[4–8] Physical activity and sedentary behaviour can be partitioned into the domains of occupation, leisure, travel and home, enabling a more nuanced exploration of how movement behaviours may impact upon health. In the travel domain, active travel, or its constituent active commuting (walking or cycling to work) have been associated with favourable health outcomes,[9–11] whilst car use may pose a risk to cardiometabolic health.[12] Physical activity and sedentary behaviour within the leisure domain, such as sport[13] and screen time[3] respectively have also been linked to health markers. Analyses of behavioural domains suggest that reallocating screen time to physical activity in the home and leisure domains (together referred to as discretionary time) reduces mortality risk.[14]

Active living approaches seek to promote physical activity and reduce sedentary time across different domains.[15] However, there is little information on how movement behaviours in different domains relate to each other. Displacement may occur within domains or between domains. Displacement can be further conceptualised as one-for-one (where time is reallocated from one domain to one other domain), one-for-multiple (where time is reallocated from one domain to a number of other domains), or one-for-remaining (where time is reallocated from one domain to all remaining domains). For example, evidence suggests that increases in active commuting are not compensated for by reductions in leisure physical activity,[16,17] and that active travel is associated with relatively higher leisure physical activity and lower screen time.[18] This preliminary research indicates that active travel is associated with a broadly health-promoting pattern of behaviour overall.

Thus, there appear to be some complex trade-offs and synergies between movement behaviours in the travel, leisure and home domains, and established relationships between movement behaviours in these domains and health. Therefore, it is important to have information on potential compensatory shuffling of activities across domains when behaviour changes within domains (such as changing commute mode from car to walking or cycling). A recent methodological development in this field is the application of compositional data analysis, which entails expressing behaviours as ratios of daily time. This allows for the exploration of the relative distribution and redistribution of behaviours.[19] Compared with traditional methods, compositional data analysis allows for the nuanced examination of the relationships between behavioural domains. It has the advantage of remaining congruent with the underlying co-dependent nature of the data, in that increases in time spent in one domain may be compensated for by reductions in time elsewhere.

Using a large epidemiological cohort, the aims of this compositional data analysis were to explore:

- The cross-sectional relationship between active commuting and the relative composition of discretionary time, incorporating recreational walking, sport and do-it-yourself (DIY) activities, and screen time
- The cross-sectional relationship between active commuting and the absolute amount of discretionary time
- The longitudinal relationship between changes in active commuting and the relative composition and absolute amount of discretionary time

## Methods

### Study population and design

UK Biobank is a large prospective cohort of British adults aged 40–69 years. Potential participants in this age group and living in proximity to one of 22 assessment centres across the country were identified from National Health Service registers. The response rate was 5.5%, with 502,633 participants attending a baseline assessment visit between March 2006 and October 2010, which included completion of an electronic touch screen questionnaire.[20] The study was approved by the North West Multi-centre Research Ethics Committee, the Patient Information Advisory Group, and the Community Health Index Advisory Group. All participants provided written informed consent. More details on the design and methods of the baseline assessment can be found elsewhere.[21,22] The first repeat assessment was carried out between December 2009 and June 2013 (n = 20,346) and the second between April 2014 and November 2016 (n = 11,923).[23] All repeat assessments included repeat administration of the electronic questionnaire and were restricted only to participants who lived near a single assessment centre in Stockport, England.

### Assessment of movement behaviours in the travel domain

At all assessments, participants who reported being self-employed or in paid employment answered questions about their mode of travel to work, with four response options: (i) car or motor vehicle; (ii) public transport; (iii) walk and (iv) cycle. Participants could select a single mode or a combination of modes. Participants also reported the weekly frequency of travel, and the distance (miles), between home and work. Adults who commuted less than once a week or for zero miles were assumed to work from home and were excluded from analysis. Those who reported not being able to walk for any reason were also excluded.

Consistent with previous analyses of active commuting using this dataset,[24,25] commute mode was dichotomised as inactive (car only) or active (any other mode or combination of modes) for the cross-sectional analysis. Public transport, walking and cycling were all considered active modes, as objective assessment confirms that all entail moderate to vigorous physical activity.[26,27] For the longitudinal analysis four categories were created: (i) car only at both observations (stable inactive); (ii) use of any other mode than car at both observations (stable active); (iii) switch from car only to any other mode (inactive to active); or (iv) switch from any other mode to car only (active to inactive).[24,25]

### Assessment of movement behaviours in the leisure and home domains (discretionary time)

At all assessments, participants answered questions about their sedentary behaviour and physical activity during discretionary time. These questions were adapted, based on piloting, from several existing validated tools.[28–30] Participants reported how many hours they spent watching television and using a computer outside of work on a typical day (open-ended question, not distinguishing between week and weekend days). These were summed to produce daily screen time and truncated at nine hours per day.[14] Estimates were converted to minutes per week.

Participants reported whether they undertook five activities during the preceding four weeks: (i) walking for pleasure (not as a means of travel); (ii) strenuous sports (described as activities that make you sweat or breathe hard); (iii) other less strenuous activities such as swimming or fitness classes; (iv) light do-it-yourself (DIY) activities such as watering the lawn; and (v) heavy DIY activities such as chopping wood or lifting heavy objects. Where participants reported having undertaken any activity, they reported the frequency and duration according to pre-specified categories (e.g. ‘2–3 times a week’ for ‘15–30 minutes’). Monthly frequencies were divided to equate to weekly frequencies and durations coded according to the mid-point of the category (e.g. 22.5 minutes for those who responded ‘15–30 minutes’). Frequency was multiplied by duration to give minutes per week in each of the five activities.

### Defining the subcomposition and total amount of discretionary time

The principles of compositional data analysis and the application of this technique to health research are described elsewhere.[19,31] A further explanation of the core features of this analytical approach with a worked example for active travel can be found in a previous analysis.[18] Compositional data are comprised of parts which sum to a whole; defining a composition entails delineating these parts and requires consideration of both the research question and the nature of the data. A subcomposition, or sub-set of parts, may also be defined where parts may be logically or conceptually grouped. This is still consistent with the nature of compositional data, as the principle of subcompositional coherence dictates that the relationship between parts is maintained regardless of the presence or absence of other parts in the analysis.[32]

The presence of zeros in any part complicates the use of compositional data analysis and a common approach is to amalgamate parts to avoid this issue. Where zeros are seen as rounded (the product of measurement imprecision rather than a true zero value, such as when categorical rather than continuous response scales are used), imputation strategies may be used to replace zeros with small non-zero numbers.[33] Because of the large number of zero values, strenuous and less strenuous sports were combined and amalgamated with light and heavy DIY activities. Screen time and walking for pleasure were seen as important standalone parts because of the potential flow of time between travel and these discretionary activities. The final three-part subcomposition consisted of:

- Screen time (minutes per week)
- Walking for pleasure (minutes per week)
- Sport and DIY activities (minutes per week)

Parts were summed to produce total discretionary time in minutes per week. This nomenclature is used for convenience and it is acknowledged that we did not comprehensively account for all discretionary time.

Zero values in the three parts were replaced with small values using a log-ratio data augmentation algorithm.[33] This algorithm requires at least one complete part; therefore, we excluded a small number of participants reporting zero screen time. We also excluded those who reported more than the equivalent of 24 hours per day of discretionary time.

### Expressing the subcomposition as sets of isometric log-ratio coordinates

Compositional data analysis can be done by expressing compositions as log-ratio coordinates, and then using the coordinates as unique variables in regression models. In essence, each log-ratio coordinate provides information on one or more parts relative to others. We use the term ‘coordinates’ in the mathematical sense to refer to the location of the composition within the sample space, which defines its value. The sample space can be understood as the set of all possible values that variables can take. Compositions exist in a sample space known as the simplex, and linear regression models are designed for a sample space known as real space. Analogous to coordinates on a geographical map, log-ratio coordinates map the composition from the simplex into real space without losing any relative information about the composition.[34]

**Fig 1:**
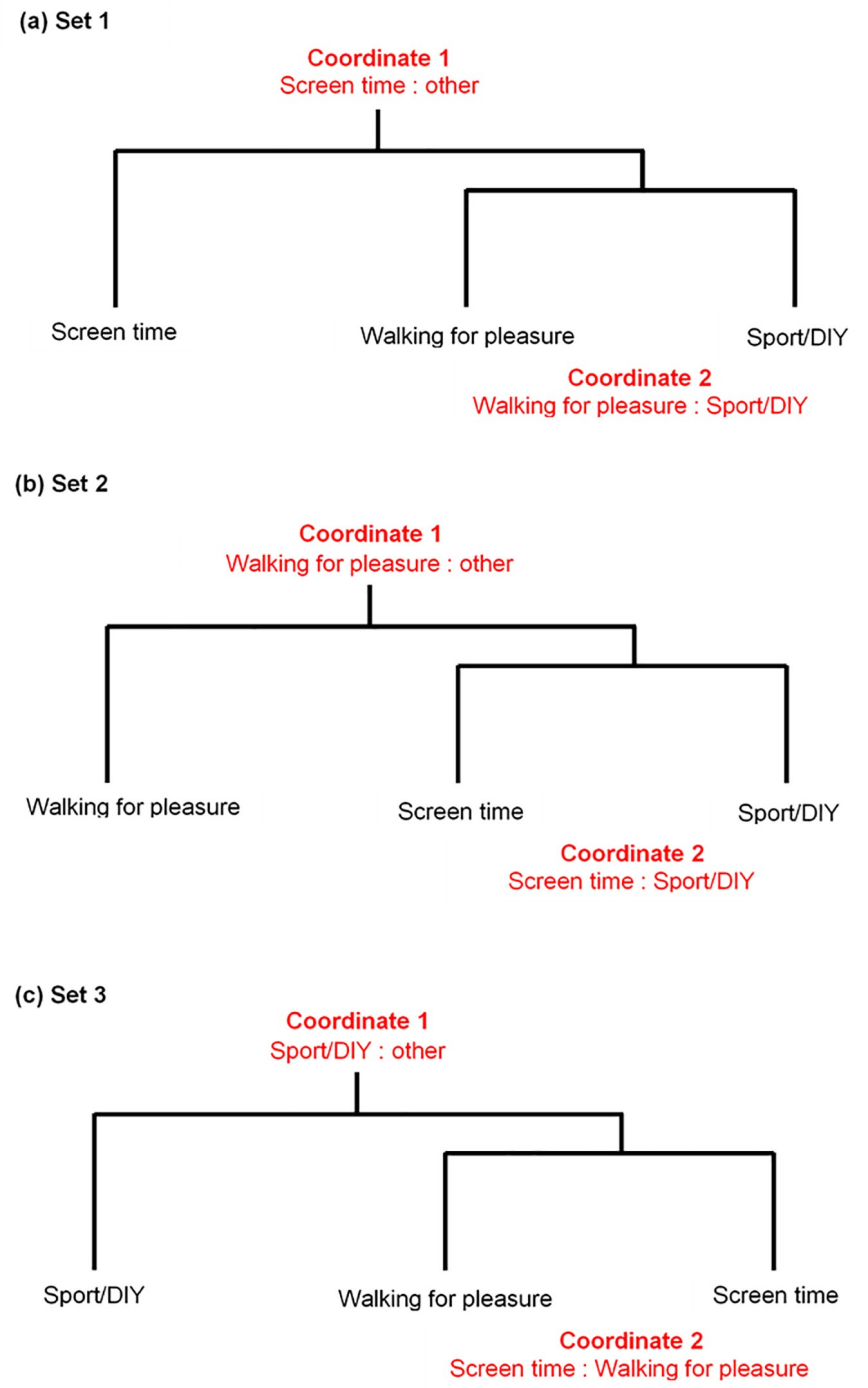
Sequential binary partition of three-part composition.

For the current analysis we used an isometric log-ratio (ilr) transformation which produces a set of coordinates numbering one less than the number of parts. From the original three-part subcomposition, we produced a set of two ilr coordinates for each participant. The set of ilr coordinates together describe the total variance of the subcomposition. Coordinates can be made interpretable through the use of sequential binary partition. This splits the composition into successively smaller groups of parts.[35,36] A tree diagram or dendrogram can be used to visualise the partitions. We used sequential binary partition to define the set, so that the first coordinate represented the ratio of screen time to the other behaviours, and the second coordinate represented the ratio of walking for pleasure to sport and DIY (Fig 1). Using the first ilr coordinate as the outcome variable in a regression model, a positive regression coefficient would indicate that the exposure variable is associated with a relatively higher level of screen time and relatively lower level of the other behaviours. In this example, the ratio is between screen time as the numerator, and the geometric mean of the remaining behaviours as the denominator.

Again using sequential binary partition, we defined two further sets of ilr coordinates for each participant. For each set, the first coordinate was expressed as one part relative to the others. In total, each participant’s subcomposition was expressed as three sets of two ilr coordinates (Fig 1). Using any of the three sets of ilr coordinates in a regression model would give identical multiple correlation coefficients, as all three sets describe the same subcomposition. However, for each set, the first coordinate gave information on one individual part relative to the rest. This allowed us to isolate and explore the dominance of each of the three parts (screen time, walking for pleasure, and sport/DIY).

### Covariates

The covariates were: weekly frequency of travel, the distance in miles between home and work, age, sex, ethnicity, home ownership, car ownership, income, education level, children in the household, Townsend score (an indicator of material deprivation calculated according to home postcode), baseline height and weight used to calculate body mass index, whether job entailed standing, walking or manual labour, bone fracture in the last five years, ever being diagnosed with a vascular condition such as a heart attack or stroke, and ever being diagnosed with a non-vascular condition such as diabetes or cancer. All covariates apart from Townsend score and body mass index were self-reported. All covariates were hypothesised to confound the association of interest (movement behaviours in the travel, leisure and home domains) based on theoretical relationships between variables and prior research.[14,25] The baseline value of all covariates was used in all models.

### Analysis

Stata 14 (College Station, TX: StataCorp LP) was used for data cleaning procedures and deriving all variables of interest. For analysis, we used the open source software R (www.r-project.org) and the bespoke packages Compositions,[37] zCompositions[33] and robCompositions.[38]

#### Cross-sectional analysis

We used baseline data from all participants who provided complete information on exposure (active commuting) and outcomes (composition and total amount of discretionary time), as well as complete information on all covariates. Using one-way ANOVA and chi-squared tests, we tested for differences between participants included in the cross-sectional analysis and the rest of the baseline sample.

We conducted an initial descriptive analysis of all variables. For the subcomposition, we calculated the arithmetic mean, median and geometric mean of each part separately. We then calculated the overall compositional mean firstly in proportions and then in minutes per week (i.e. as proportions of the mean of total discretionary time).

The analysis consisted of three stages. First, we tested for an association between commute mode (inactive vs active) and one of the sets of ilr coordinates representing the discretionary time subcomposition. We used linear regression models (MANCOVA) progressively adjusted for the covariates described above. The MANCOVA findings would be identical using any of the three sets of ilr coordinates. This analysis gave an indication of whether the discretionary time subcomposition differed overall between those who used active travel modes and those who used inactive travel modes. However, it did not identify which particular parts (i.e. screen time, walking for pleasure, or sport/DIY) were driving this difference.

Second, we tested for an association between commute mode (inactive vs active) and the first coordinate of each of the three sets of ilr coordinates (Fig 1) using adjusted linear regression models. This gave an indication of whether screen time, walking for pleasure, or sport/DIY differed between those who used active travel modes and those who used inactive travel modes, relative to the other parts.

Third, we tested for an association between commute mode (inactive vs active) and total discretionary time (log transformed to satisfy the assumption that the variable can take both positive and negative values), using adjusted linear regression models. This gave an indication of whether total discretionary time was associated with travel modes, regardless of the composition of discretionary time. In all regression models, visual inspection of the distribution of residuals indicated that the assumptions for linear regression had been met.

Finally, we used the models to predict adjusted compositional means for those who used active travel modes and those who used inactive travel modes. Using the R package lsmeans,[39] we estimated the adjusted mean ilr coordinate value for each of the two ilr coordinates comprising a set. Subsequently, we back-transformed the ilr set using the same ilr partitioning system, firstly into proportions. We then transformed the proportions into minutes per week based on the adjusted mean value of total discretionary time in those who used active travel modes and those who used inactive travel modes separately.

#### Longitudinal analysis

We used data from all participants who provided complete information on exposure, outcomes and covariates at baseline and first repeat assessment. We tested for differences between participants included in the longitudinal analysis and participants included in the cross-sectional analysis.

We followed the same general approach described for the cross-sectional analysis. We conducted an initial descriptive analysis of all variables. We then tested for associations between change in commute mode (stable inactive, stable active, inactive to active, active to inactive) and (i) change in the overall discretionary time subcomposition; (ii) relative changes in screen time, walking for pleasure, and sport/DIY; and (iii) change in total discretionary time. We used linear regression models progressively adjusted for the baseline value of the covariates described. Additionally, we derived a continuous variable of the time elapsed between baseline and first repeat assessment, defined according to the dates of assessment; and a variable indicating whether the season differed between assessments. We used these two variables as additional covariates in the maximally adjusted models. For all outcomes, we used the follow-up value adjusted for the baseline value to represent change over time. In all regression models, visual inspection of the distribution of residuals indicated that the assumptions for linear regression had been met. Finally, we used the models to predict adjusted compositional means for the different commute categories.

#### Sensitivity analyses

Because of the large amount of missing data on income and number of children in the household, we repeated the cross-sectional and longitudinal analyses removing these covariates, which markedly increased the sample size.

## Results

From an initial sample of 502,617 participants who provided baseline data, we firstly limited the sample to 246,110 participants who were employed, worked outside the home, reported a commute mode and were able to walk. Following that, we limited the sample to 243,954 participants who had reported some discretionary and screen time, but not more than 24 hours per day of discretionary time. We then limited the sample to those providing complete information on all covariates, leaving a final cross-sectional sample of 182,406 participants (36% of the initial sample).

From an initial sample of 20,346 participants who provided information at both baseline and first repeat assessment, the longitudinal sample was firstly limited to 6,201 participants who provided complete information on exposures, then to 6,133 participants who provided complete information on outcomes, with the final sample limited to 4,323 participants who additionally provided complete information on covariates (21% of the initial sample). The baseline characteristics of the cross-sectional and longitudinal samples are shown in Table 1.

**Table 1:** Baseline characteristics of the analysis samples.

Variable	Cross-sectional sample (n = 182,406)	Longitudinal sample (n = 4,323)
	Mean (SD) or n (%)	Mean (SD) or n (%)
Commute mode		
Inactive	118,569 (65.0)	3,131 (72.4)
Active	63,837 (35.0)	1,192 (27.6)
Commute frequency (outward trips/week)	4.6 (1.7)	4.6 (1.4)
Distance between home and work (miles)	12.0 (36.2)	13.5 (31.8)
Age (years)	52.2 (6.9)	50.8 (6.1)
Sex		
Male	89,946 (49.3)	2,186 (50.6)
Female	92,460 (50.7)	2,137 (49.4)
Ethnicity		
White	173,057 (94.9)	4,202 (97.2)
Mixed	1,154 (0.6)	21 (0.5)
Asian	3,521 (1.9)	43 (1.0)
Black	2,665 (1.5)	27 (0.6)
Chinese	626 (0.3)	13 (0.3)
Other	1,383 (0.8)	17 (0.4)
Home ownership		
Owner-occupier	171,134 (93.8)	4,205 (97.3)
Other (e.g. rents)	11,272 (6.2)	118 (2.7)
Car ownership		
Owns at least one car	176,426 (96.7)	4,270 (98.8)
Does not own a car	5,980 (3.3)	53 (1.2)
Household income		
<£18,000	12,179 (6.7)	160 (3.7)
£18,000–30,999	34,578 (19.0)	651 (15.1)
£31,000–51,999	59,688 (32.7)	1,399 (32.4)
£52,000–100,000	60,268 (33.0)	1,702 (39.4)
>£100,000	15,693 (8.6)	411 (9.5)
Education level		
University or college degree	71,115 (39.0)	2,118 (49.0)
Further education	22,987 (12.6)	607 (14.0)
Higher secondary education	39,892 (21.9)	820 (19.0)
Secondary education	12,918 (7.1)	223 (5.2)
Vocational qualifications	12,378 (6.8)	258 (6.0)
Other professional qualifications	7,624 (4.2)	160 (3.7)
None of the above	15,492 (8.5)	137 (3.2)
Has at least one child		
Yes	175,466 (96.2)	4,173 (96.5)
No	6,940 (3.8)	150 (3.5)
Townsend score^a^	-1.7 (2.8)	-2.1 (2.6)
Body mass index (kg/m^2^)	27.2 (4.6)	26.6 (4.4)
Work physical activity		
Job involves mostly sitting	120,122 (65.9)	3,164 (73.2)
Job involves mostly standing or manual labour	62,284 (34.2)	1,159 (26.8)
Bone fracture in the preceding five years		
Yes	15,744 (8.6)	320 (7.4)
No	166,662 (91.4)	4,003 (92.6)
Non-vascular condition or disability^b^		
Yes	38,330 (21.0)	850 (19.7)
No	144,076 (79.0)	3,473 (80.3)
Vascular condition^c^		
Yes	39,542 (21.7)	680 (15.7)
No	142,864 (78.3)	3,643 (84.3)

kg–kilogram; m–metre; n–number; SD–standard deviation

There were no missing data on any of the variables of interest as the samples were restricted to those with complete data

^a^Range from -6.3 to 11.0, where higher scores indicate higher levels of deprivation

^b^Defined according to whether participants reported ever receiving a doctor’s diagnosis for diabetes, cancer or 'any other serious medical conditions or disabilities'

^c^Defined according to whether participants reported ever receiving a doctor’s diagnosis for angina, heart attack, high blood pressure or stroke

For the sensitivity analysis, the sample size was 237,036 for the cross-sectional analysis (47% of the initial sample) and 5,967 for the longitudinal analysis (29% of the initial sample).

### Cross-sectional analysis

#### Descriptive analysis

The cross-sectional sample were predominantly middle-aged, White and high socioeconomic status across a range of factors (education, income, home ownership and car ownership). Two thirds (65%) commuted by inactive modes (car only) at baseline. The cross-sectional sample differed from the rest of the baseline sample in all demographic and health characteristics, consistent with an employed population that was the focus of this analysis (S1 Table).

Descriptive characteristics of the raw subcomposition (including zero values) and the imputed subcomposition (in which small numbers were imputed in place of zero values) can be found in Table 2. Screen time comprised the vast majority (95%) of discretionary time, with a compositional mean of 1635 minutes per week or approximately four hours per day.

**Table 2:** Descriptive characteristics of the discretionary time subcomposition in the cross-sectional sample (n = 182,406).

Part	Raw composition		Imputed composition		
	*Arithmetic mean (SD) in minutes/week*	*Median (IQR) in minutes/week*	*Geometric mean in minutes/week*	*Compositional mean in proportions*	*Compositional mean in minutes/week*^*a*^
Screen time	1469 (796)	1260 (840–1680)	1270	0.95	1635
Walking for pleasure	78 (124)	28 (0–105)	18	0.01	23
Sport and DIY activities	176 (263)	94 (23–218)	51	0.04	66

DIY—do-it-yourself; IQR–interquartile range; SD–standard deviation

There were no missing data on any of the variables of interest as the sample was restricted to those with complete data

^a^Based on the mean of total discretionary time for the entire sample (1724 minutes/week)

#### Association between commute mode and the discretionary time subcomposition

In all models, there was a statistically significant (p<0.01) association between commute mode and the set of two ilr coordinates. This indicated that the composition of discretionary time differed overall between those who used active travel modes and those who used inactive travel modes.

#### Association between commute mode and screen time, walking for pleasure and sport/DIY

In all models, there was a statistically significant (p<0.01) association between commute mode and the ilr of screen time, relative to the other activities (Table 3). A negative coefficient indicated that compared to inactive modes, those who used active modes of travel engaged in less screen time relative to the other activities.

**Table 3:** Cross-sectional association between commute mode and screen time, walking for pleasure, sport/DIY and total discretionary time (n = 182,406).

Part	Beta coefficient (95% CI)		
	*Model 1*	*Model 2*	*Model 3*
Screen time : rest^a^	-0.12 (-0.13, -0.10)	-0.16 (-0.17, -0.14)	-0.12 (-0.13,– 0.11)
Walking for pleasure : rest^a^	0.15 (0.13, 0.17)	0.12 (0.10, 0.14)	0.10 (0.08, 0.12)
Sport and DIY activities : rest^a^	-0.03 (-0.05, -0.01)	0.04 (0.02, 0.06)	0.02 (0.00, 0.04)
Total discretionary time	-0.07 (-0.07, -0.06)	-0.05 (-0.05, -0.04)	-0.04 (-0.04, -0.03)

CI–confidence interval; DIY—do-it-yourself

^a^Coefficients are for active travel mode with inactive travel mode as the reference category. A positive coefficient indicates that those who used active modes of travel engaged in more of that part relative to the other activities, and a negative coefficient indicates that those who used active modes of travel engaged in less of that part relative to the other activities

Model 1 is unadjusted

Model 2 is adjusted for weekly frequency of travel, distance in miles between home and work, age, sex, ethnicity, home ownership, car ownership, income, education level, children in the household and Townsend score

Model 3 is adjusted for the covariates in Model 2 plus body mass index, whether job entailed standing, walking or manual labour, bone fracture in the last five years, ever being diagnosed with a vascular condition and ever being diagnosed with a non-vascular condition

In all models, there was a statistically significant (p<0.01) association between commute mode and the ilr of walking for pleasure, relative to the other activities (Table 3). A positive coefficient indicated that compared to inactive modes, those who used active modes of travel spent more time walking for pleasure relative to the other activities.

In all models, there was a statistically significant (p<0.05) association between commute mode and the ilr of sport/DIY, relative to the other activities (Table 3). The coefficients were small, and while statistically significant, did not indicate a strong or consistent relationship.

#### Association between commute mode and total discretionary time

In all models, there was a statistically significant (p<0.01) association between commute mode and total discretionary time (Table 3). A negative coefficient indicated that total discretionary time was lower in those who used active travel modes compared to those who used inactive modes.

#### Model-predicted compositional mean and total discretionary time

Model-predicted weekly time spent in the three discretionary activities and total weekly discretionary time are presented in Table 4. Because of the difference between groups in total discretionary time, the absolute differences between groups in the parts comprising discretionary time were small. Those who used active modes engaged in approximately one hour less screen time, and one hour less total discretionary time, per week than those who used inactive modes.

**Table 4:** Model-predicted compositional mean and total discretionary time in the cross-sectional sample (n = 182,406).

Part	Active commute mode	Inactive commute mode
	*Compositional mean in minutes/week*^*a*^	*Compositional mean in minutes/week*^*a*^
Screen time	1589	1653
Walking for pleasure	10	8
Sport and DIY activities	18	17
Total discretionary time	1616	1678

^a^Based on the maximally adjusted model-predicted mean of total discretionary time for active and inactive travel modes separately

### Longitudinal analysis

#### Descriptive analysis

The longitudinal sample differed significantly from the rest of the cross-sectional sample in all of the variables listed in Table 1 apart from sex, children in the household and weekly frequency of commuting. Again, the longitudinal sample were predominantly middle-aged, White and high socioeconomic status, and even more so than the cross-sectional sample. Nearly three quarters (72%) commuted by inactive modes (car only) at baseline. Of the 4,323 participants included in analysis, 2,783 (64%) were stable inactive (i.e. car only at both observations), 902 (21%) were stable active (i.e. use of any other mode than car at both observations), 348 (8%) switched from inactive to active modes, and 290 (7%) switched from active to inactive modes. On average, the time elapsed between assessments was 4.3 (standard deviation [SD] 0.9) years. 73% of participants completed the follow-up assessment in a different season to the baseline assessment.

Descriptive characteristics of the raw composition (including zero values) and the imputed composition (in which small numbers were imputed in place of zero values) at baseline and follow-up can be found in Table 5. Screen time and walking for pleasure increased over time, whereas sport and DIY activities decreased.

**Table 5:** Descriptive characteristics of the discretionary time subcomposition in the longitudinal sample (n = 4,323).

Part	Raw composition		Imputed composition		
	*Arithmetic mean (SD) in minutes/week*	*Median (IQR) in minutes/week*	*Geometric mean in minutes/week*	*Compositional mean in proportions*	*Compositional mean in minutes/week*^*a*^
*Baseline*					
Screen time	1345 (740)	1260 (840–1680)	1162	0.93	1500
Walking for pleasure	73 (110)	28 (0–105)	18	0.01	23
Sport and DIY activities	194 (259)	113 (34–248)	69	0.05	89
*Follow-up*					
Screen time	1533 (795)	1260 (1050–1890)	1343	0.95	1689
Walking for pleasure	81 (121)	45 (0–113)	20	0.01	25
Sport and DIY activities	173 (239)	103 (28–225)	58	0.04	72

DIY—do-it-yourself; IQR–interquartile range; SD–standard deviation

There were no missing data on any of the variables of interest as the sample was restricted to those with complete data

^a^Based on the mean of total discretionary time for the entire sample (1612 minutes/week at baseline; 1787 minutes/week at follow-up)

#### Association between change in commute mode and change in the discretionary time subcomposition

In all models, there was a statistically significant (p<0.05) association between change in commute mode and change in the set of two ilr coordinates. This indicated that the composition of discretionary time changed differentially over time among the travel mode change categories (stable inactive, stable active, inactive to active, active to inactive).

#### Association between change in commute mode and change in screen time, walking for pleasure and sport/DIY

In all models, there was a statistically significant (p<0.01) association between change in commute mode and change in the ilr of screen time, relative to the other activities (Table 6). A negative coefficient indicated that compared to those who used inactive modes at both time points, those who used active modes of travel at both time points reduced their screen time relative to the other activities. However, no differences were seen for individuals who changed their mode of travel over time (i.e. inactive to active or active to inactive).

**Table 6:** Longitudinal association between travel mode and screen time, walking for pleasure, sport/DIY and total discretionary time (n = 4,323).

Part	Beta coefficient (95% CI)		
	*Model 1*	*Model 2*	*Model 3*
*Screen time* : *rest*^a^			
stable inactive	ref	ref	ref
stable active	-0.15 (-0.23, -0.06)	-0.17 (-0.26, -0.09)	-0.15 (-0.24, -0.06)
inactive to active	0.02 (-0.11, 0.15)	0.02 (-0.10, 0.14)	0.02 (-0.10, 0.15)
active to inactive	-0.04 (-0.18, 0.09)	-0.05 (-0.19, 0.09)	-0.05 (-0.19, 0.09)
*Walking for pleasure* : *rest*^a^			
stable inactive	ref	ref	ref
stable active	0.19 (0.06, 0.32)	0.19 (0.05, 0.32)	0.18 (0.04, 0.31)
inactive to active	0.03 (-0.16, 0.22)	0.00 (-0.19, 0.19)	0.00 (-0.19, 0.19)
active to inactive	0.01 (-0.20, 0.21)	0.00 (-0.20, 0.21)	0.00 (-0.20, 0.21)
*Sport and DIY activities* : *rest*^a^			
stable inactive	ref	ref	ref
stable active	-0.01 (-0.13, 0.11)	0.02 (-0.11, 0.15)	-0.01 (-0.14, 0.12)
inactive to active	-0.01 (-0.19, 0.17)	0.02 (-0.17, 0.20)	0.01 (-0.17, 0.19)
active to inactive	0.06 (-0.13, 0.26)	0.06 (-0.13, 0.26)	0.07 (-0.13, 0.26)
*Total discretionary time*			
stable inactive	ref	ref	ref
stable active	-0.03 (-0.06, 0.00)	-0.03 (-0.06, 0.00)	-0.02 (-0.05, 0.01)
inactive to active	0.03 (-0.01, 0.07)	0.03 (-0.01, 0.07)	0.03 (-0.01, 0.07)
active to inactive	-0.03 (-0.07, 0.02)	-0.02 (-0.07, 0.02)	-0.02 (-0.07, 0.02)

CI–confidence interval; DIY—do-it-yourself

^a^Coefficients are for a particular commute category with stable inactive as the reference category. A positive coefficient indicates that those in a particular commute category engaged in more of that part relative to the other activities, and a negative coefficient indicates that those in a particular commute category travel engaged in less of that part relative to the other activities

Model 1 is unadjusted

Model 2 is adjusted for weekly frequency of travel, distance in miles between home and work, age, sex, ethnicity, home ownership, car ownership, income, education level, children in the household and Townsend score

Model 3 is adjusted for the covariates in Model 2 plus body mass index, whether job entailed standing, walking or manual labour, bone fracture in the last five years, ever being diagnosed with a vascular condition, ever being diagnosed with a non-vascular condition, time elapsed between assessments and whether the season differed between assessments

In all models, there was a statistically significant (p<0.01) association between change in commute mode and change in the ilr of walking for pleasure, relative to the other activities (Table 6). A positive coefficient indicated that compared to those who used inactive modes at both time points, those who used active modes of travel at both time points increased walking for pleasure relative to the other activities. However, no differences were seen for individuals who changed their mode of travel over time (i.e. inactive to active or active to inactive).

In all models, there were no statistically significant associations between change in commute mode and change in the ilr of sport/DIY, relative to the other activities (Table 6).

#### Association between change in commute mode and change in total discretionary time

In the unadjusted model, there was a statistically significant (p<0.05) association between change in commute mode and change in total discretionary time (Table 6). A negative coefficient indicated that compared to those who used inactive modes at both time points, those who used active modes of travel at both time points reduced their total discretionary time. However, this association was not statistically significant in the maximally adjusted model.

#### Model-predicted compositional mean and total discretionary time

Model-predicted weekly time spent in the three discretionary activities and total weekly discretionary time at follow up are presented in Table 7. The absolute differences between groups in the parts comprising discretionary time were small. At follow-up, those who used active modes at both time points engaged in approximately half an hour less screen time per week than those who used inactive modes at both time points.

**Table 7:** Model-predicted compositional mean and total discretionary time at follow-up in the longitudinal sample (n = 4,323).

Part	Stable inactive	Stable active	Inactive to active	Active to inactive
	*Compositional mean in minutes/week*^*a*^	*Compositional mean in minutes/week*^*a*^	*Compositional mean in minutes/week*^*a*^	*Compositional mean in minutes/week*^*a*^
Screen time	1660	1623	1710	1617
Walking for pleasure	15	19	15	15
Sport and DIY activities	45	49	46	48
Total discretionary time	1720	1691	1771	1680

^a^Based on the maximally adjusted model-predicted mean of total discretionary time for each commute category separately

### Sensitivity analyses

The cross-sectional and longitudinal sensitivity analyses indicated the same pattern of findings as the main analysis (S2 and S3 Tables), with the exception that the finding on change in total discretionary time remained statistically significant in the maximally adjusted model in the longitudinal sensitivity analysis.

## Discussion

### Main findings

Overall, active commuting was associated with patterns of movement behaviour during discretionary time that appear favourable to health. In longitudinal analyses, maintenance of active commuting over an average follow-up of four years was associated with a relative reduction in screen time and increase in walking for pleasure. However, after accounting for varying amounts of total discretionary time, the absolute differences between groups were very small. The largest differences were found for screen time; those using active modes engaged in 30–60 minutes less screen time per week than those using inactive modes. Though modest, this represents a relative energy deficit of approximately 22.5 metabolic equivalent (MET) minutes per week or 0.15% of total daily energy expenditure, and could have a cumulative effect on health over time.

The findings from the cross-sectional analysis indicated a tendency for people who were physically active in one domain (travel) to also spend more time being active in others (leisure and home). However, it was notable in the longitudinal analysis (where we could explore behaviour change over time at the individual level) that changing commute mode from inactive to active or vice versa was not associated with corresponding changes to the composition of discretionary time. It may be that these changes predominantly draw time from within the travel domain, or from domains other than leisure and home. This was supported by the finding that changes in commute mode were not associated with changes in total discretionary time. Others[40] have suggested that discretionary time might be a flexible pool that could be drawn upon to accommodate behaviour change, but we found no evidence of within-domain transfers of time.

### Strength and limitations

There is burgeoning interest in the application of compositional data analysis to health research. To our knowledge, this is the first study exploring the longitudinal relationship between change in commute mode and relative changes in both physical activity and sedentary behaviour. The strengths of the study include the large sample and detailed characterisation of covariates that are a feature of the UK Biobank study. In addition, we examined movement behaviours at the domain level, in order to provide insight on whether and how behaviours redistribute between domains.

We also acknowledge the study limitations. UK Biobank is not representative of the UK general population, with a low response rate, evidence of a ‘healthy volunteer’ selection bias,[41] and the associated likelihood that heathier volunteers also display healthier patterns of movement behaviour. In addition, the longitudinal sample was drawn from only one geographical area. Using the self-reported data available, we were not able to account for all daily activities and therefore construct a complete composition; instead, we focussed on a subcomposition of specific activities occurring during discretionary time. Self-report may be subject to recall and social desirability biases, but was necessary in order to explore behaviour at the domain level. The questions used to capture activities in this study had different reference time frames (a typical day for screen time, and the preceding four weeks for the other activities), although it is unlikely this would have changed the results.[28] During data cleaning, we were required to make estimations and decisions about truncating values, though we have been transparent about these decisions and the basis for them. The longitudinal analysis was limited by the small proportion of the sample who changed travel mode; less than 10% of the sample switched from inactive to active modes or vice versa, which may have limited statistical power to detect changes in outcomes. In addition, it is likely there were other influences on discretionary time over the average four year follow-up period. Finally, as exposures and outcomes, and changes in them, were measured concurrently, reverse causation is possible.

### Comparison with previous work

The findings reported here are consistent with a previous compositional data analysis in a sample of UK adults which indicated that those who did active travel reported relatively less screen time and more leisure physical activity than those not undertaking active travel.[18] We also found some concordance with previous research suggesting that active travel was not associated with reductions in physical activity in the leisure domain.[16,17]

### Implications for research

Avenues for future research include replicating these analyses in different settings and in more representative samples to explore the generalisability of findings. In particular, individual-level longitudinal analyses using time-use diaries to assess all daily time (i.e. the full composition) would allow for a comprehensive exploration of how behavioural domains change over time. Longitudinal research should employ multiple follow-ups if possible, to explore how associations change over the shorter and longer term. Intervention research should consider the potential for one-for-one, one-for-multiple, or one-for-remainder displacement, and explore the effects of interventions on compositions rather than single behaviours.

### Implications for policy and practice

Increasing active travel is a focus of transport policy in the UK,[42] and is seen as desirable on public health grounds as a means to equitably promote population physical activity while improving traffic safety and reducing air pollution and degradation of the environment.[43,44] This analysis further indicates that lower screen time may be an additional population co-benefit of active travel.[45]

### Conclusions

In conclusion, we found that active commuting was associated with relatively less screen-based sedentary behaviour during discretionary time. Future longitudinal and intervention research should explore how behavioural domains relate to each other and change over time.

## Supporting information

S1 Table Baseline characteristics of the cross-sectional sample and the baseline sample not included in analysis. (DOCX)

S2 Table Sensitivity analysis for cross-sectional association between commute mode and screen time, walking for pleasure, sport/DIY and total discretionary time (n = 237,036). (DOCX)

S3 Table Sensitivity analysis for longitudinal association between commute mode and screen time, walking for pleasure, sport/DIY and total discretionary time (n = 5,967). (DOCX)

S4 Table Biobank variables comprising core analysis dataset. (DOCX)

S1 File STROBE checklist. (DOC)

## References

[1] Lee IM, Shiroma EJ, Lobelo F, Puska P, Blair SN, Katzmarzyk PT, et al (2012) Effect of physical inactivity on major non-communicable diseases worldwide: an analysis of burden of disease and life expectancy. The Lancet 380: 219–229. 10.1016/S0140-6736(12)61031-9 PMC3645500 22818936

[2] Biswas A, Oh PI, Faulkner GE, Bajaj RR, Silver MA, Mitchell MS, et al (2015) Sedentary time and its association with risk for disease incidence, mortality, and hospitalization in adults: a systematic review and meta-analysis. Annals of Internal Medicine 162: 123–132. 10.7326/M14-1651 25599350

[3] Ekelund U, Steene-Johannessen J, Brown WJ, Fagerland MW, Owen N, Powell KE, et al (2016) Does physical activity attenuate, or even eliminate, the detrimental association of sitting time with mortality? A harmonised meta-analysis of data from more than 1 million men and women. Lancet 388: 1302–1310. 10.1016/S0140-6736(16)30370-1 27475271

[4] Buman MP, Winkler EA, Kurka JM, Hekler EB, Baldwin CM, Owen N, et al (2014) Reallocating time to sleep, sedentary behaviors, or active behaviors: associations with cardiovascular disease risk biomarkers, NHANES. American Journal of Epidemiology 179: 323–334. 10.1093/aje/kwt292 24318278

[5] Hamer M, Stamatakis E, Steptoe A (2014) Effects of substituting sedentary time with physical activity on metabolic risk. Medicine & Science in Sports & Exercise 46: 1946–1950. 24674977 10.1249/MSS.0000000000000317 PMC4186723

[6] Stamatakis E, Rogers K, Ding D, Berrigan D, Chau J, Hamer M, et al (2015) All-cause mortality effects of replacing sedentary time with physical activity and sleeping using an isotemporal substitution model: a prospective study of 201,129 mid-aged and older adults. International Journal of Behavioral Nutrition and Physical Activity 12: 121 10.1186/s12966-015-0280-7 26419654 PMC4589071

[7] van der Berg JD, van der Velde JHPM, De Waard EAC, Bosma H, Savelberg HHCM, Schaper NC, et al (2017) Replacement effects of sedentary time on metabolic outcomes: the Maastricht study. Medicine & Science in Sports & Exercise 49: 1351–1358. 28263284 10.1249/MSS.0000000000001248

[8] Chastin SFM, Palarea-Albaladejo J, Dontje ML, Skelton DA (2015) Combined effects of time spent in physical activity, sedentary behaviors and sleep on obesity and cardio-metabolic health markers: a novel compositional data analysis approach. PLoS ONE 10: e0139984 10.1371/journal.pone.0139984 26461112 PMC4604082

[9] Kelly P, Kahlmeier S, Götschi T, Orsini N, Richards J, Roberts N, et al (2014) Systematic review and meta-analysis of reduction in all-cause mortality from walking and cycling and shape of dose response relationship. International Journal of Behavioral Nutrition and Physical Activity 11: 132 10.1186/s12966-014-0132-x 25344355 PMC4262114

[10] Celis-Morales CA, Lyall DM, Welsh P, Anderson J, Steell L, Guo Y, et al (2017) Association between active commuting and incident cardiovascular disease, cancer, and mortality: prospective cohort study. British Medical Journal 357: j1456 10.1136/bmj.j1456 28424154

[11] Panter J, Mytton O, Sharp S, Brage S, Cummins S, Laverty AA, et al (2018) Using alternatives to the car and risk of all-cause, cardiovascular and cancer mortality. Heart 10.1136/heartjnl-2017-312699 29785956 PMC6241630

[12] Sugiyama T, Wijndaele K, Koohsari MJ, Tanamas SK, Dunstan DW, Owen N. (2016) Adverse associations of car time with markers of cardio-metabolic risk. Preventive Medicine 83: 26–30. 10.1016/j.ypmed.2015.11.029 26656405 PMC5405044

[13] Samitz G, Egger M, Zwahlen M (2011) Domains of physical activity and all-cause mortality: systematic review and dose–response meta-analysis of cohort studies. International Journal of Epidemiology 40: 1382–1400. 10.1093/ije/dyr112 22039197

[14] Wijndaele K, Sharp SJ, Wareham NJ, Brage S (2017) Mortality risk reductions from substituting screen time by discretionary activities. Medicine & Science in Sports & Exercise 49: 1111–1119. 28106621 10.1249/MSS.0000000000001206 PMC5402872

[15] Public Health England (2017) Everybody active, every day: two years on. An update on the national physical activity framework London: Public Health England.

[16] Sahlqvist S, Goodman A, Cooper AR, Ogilvie D (2013) Change in active travel and changes in recreational and total physical activity in adults: Longitudinal findings from the iConnect study. International Journal of Behavioral Nutrition and Physical Activity 10: 28 10.1186/1479-5868-10-28 23445724 PMC3598920

[17] Foley L, Panter J, Heinen E, Prins RG, Ogilvie D (2015) Changes in active commuting and changes in physical activity in adults: a cohort study. International Journal of Behavioral Nutrition and Physical Activity 12: 161 10.1186/s12966-015-0323-0 26682539 PMC4683976

[18] Foley L, Dumuid D, Atkin AJ, Olds T, Ogilvie D (2018) Patterns of health behaviour associated with active travel: a compositional data analysis. International Journal of Behavioral Nutrition and Physical Activity 15: 26 10.1186/s12966-018-0662-8 29562923 PMC5861598

[19] Dumuid D, Stanford TE, Martin-Fernández JA, Pedišić Ž, Maher C, Lewis LK, et al (2017) Compositional data analysis for physical activity, sedentary time and sleep research. Statistical Methods in Medical Research 10.1177/0962280217710835. 28555522

[20] Biobank UK (2011) UK Biobank touchscreen questionnaire. Stockport, UK: UK Biobank.

[21] Allen N, Sudlow C, Downey P, Peakman T, Danesh J, Elliott P, et al (2012) UK Biobank: current status and what it means for epidemiology. Health Policy and Technology 1: 123–126.

[22] Biobank UK (2007) Protocol for a Large-Scale Prospective Epidemiological Resource. Stockport, UK: UK Biobank.

[23] Biobank UK (2013) Repeat Assessment Data: September 2013. Version 1.0. Stockport, UK: UK Biobank.

[24] Flint E, Webb E, Cummins S (2016) Change in commute mode and body-mass index: prospective, longitudinal evidence from UK Biobank. Lancet Public Health 1: e46–e55. 10.1016/S2468-2667(16)30006-8 28299370 PMC5341146

[25] Knott CS, Panter J, Foley L, Ogilvie D (2018) Changes in the mode of travel to work and the severity of depressive symptoms: a longitudinal analysis of UK Biobank. Preventive Medicine 112: 61–69. 10.1016/j.ypmed.2018.03.018 29604327 PMC5999356

[26] Ainsworth BE, Haskell WL, Herrmann SD, Meckes N, Bassett DRJ, Tudor-Locke C, et al (2011) 2011 Compendium of Physical Activities: a second update of codes and MET values. Medicine & Science in Sports & Exercise 43: 1575–1581. 21681120 10.1249/MSS.0b013e31821ece12

[27] Costa S, Ogilvie D, Dalton A, Westgate K, Brage S, Panter J. (2015) Quantifying the physical activity energy expenditure of commuters using a combination of global positioning system and combined heart rate and movement sensors. Preventive Medicine 81: 339–344. 10.1016/j.ypmed.2015.09.022 26441297 PMC4678256

[28] Craig CL, Marshall AL, Sjostrom M, Bauman AE, Booth ML, Ainsworth BE, et al (2003) International Physical Activity Questionnaire: 12-country reliability and validity. Medicine & Science in Sports & Exercise 35: 1381–1395. 12900694 10.1249/01.MSS.0000078924.61453.FB

[29] Hu FB, Li TY, Colditz GA, Willett WC, Manson JE (2003) Television watching and other sedentary behaviors in relation to risk of obesity and type 2 diabetes mellitus in women. Journal of the American Medical Association 289: 1785–1791. 10.1001/jama.289.14.1785 12684356

[30] Jakes RW, Day NE, Khaw KT, Luben R, Oakes S, Welch A, et al (2003) Television viewing and low participation in vigorous recreation are independently associated with obesity and markers of cardiovascular disease risk: EPIC-Norfolk population-based study. European Journal of Clinical Nutrition 57: 1089–1096. 10.1038/sj.ejcn.1601648 12947427

[31] Pedišić Ž (2017) Integrating sleep, sedentary behaviour, and physical activity research in the emerging field of time-use epidemiology: definitions, concepts, statistical methods, theoretical framework, and future directions. Kinesiology 49.

[32] Aitchison J (2003) A concise guide to compositional data analysis. Laboratório de Estatística e Geoinformação.

[33] Palarea-Albaladejo J, Martin-Fernández JA (2015) zCompositions—R package for multivariate imputation of left-censored data under a compositional approach. Chemometrics and Intelligent Laboratory Systems 143: 85–96.

[34] Mateu-Figueras G, Pawlowsky-Glahn V, Egozcue J (2011) The Principle of Working on Coordinates In: Pawlowsky-Glahn V, Buccianti A, editors. Compositional Data Analysis: Theory and Applications. Chichester, UK: John Wiley & Sons pp. 29–42.

[35] Pawlowsky-Glahn V, Egozcue JJ (2011) Exploring compositional data with the CoDa-dendrogram. Austrian Journal of Statistics 40: 103–113.

[36] Ferrer-Rosell B, Coenders G, Mateu-Figueras G, Pawlowsky-Glahn V (2016) Understanding low-cost airline users' expenditure patterns and volume. Tourism Economics 22: 269–291.

[37] van den Boogaart KG, Tolosana-Delgado R (2008) “Compositions”: a unified R package to analyze compositional data. Computers & Geosciences 34: 320–338.

[38] Templ M, Hron K, Filzmoser P (2011) robCompositions: an R-package for robust statistical analysis of compositional data In: Pawlowsky-Glahn V, Buccianti A, editors. Compositional Data Analysis: Theory and Applications. Chichester, UK: John Wiley & Sons, Ltd.

[39] Lenth RV (2016) Least-square means: the R package lsmeans. Journal of Statistical Software 69: 1–33.

[40] Gomersall SR, Norton K, Maher C, English C, Olds T (2015) In search of lost time: When people undertake a new exercise program, where does the time come from? A randomized controlled trial. Journal of Science and Medicine in Sport 18: 43–48. 10.1016/j.jsams.2014.01.004 24602689

[41] Biobank UK (2017) Access matter: representativeness of the UK Biobank resource Stockport, UK: UK Biobank.

[42] Department for Transport (2017) Cycling and walking investment strategy London, United Kingdom: Department for Transport.

[43] Sallis JF, Spoon C, Cavill N, Engelberg JK, Gebel K, Parker M, et al (2015) Co-benefits of designing communities for active living: an exploration of literature. International Journal of Behavioral Nutrition and Physical Activity 12: 30 10.1186/s12966-015-0188-2 25886356 PMC4349686

[44] Woodcock J, Banister D, Edwards P, Prentice AM, Roberts I (2007) Energy and transport. The Lancet 370: 1078–1088. 10.1016/S0140-6736(07)61254-9 17868817

[45] Patterson R, McNamara E, Tainio M, de Sá TH, Smith AD, Sharp SJ, et al (2018) Sedentary behaviour and risk of all-cause, cardiovascular and cancer mortality, and incident type 2 diabetes: a systematic review and dose response meta-analysis. European Journal of Epidemiology 33: 811–829. 10.1007/s10654-018-0380-1 29589226 PMC6133005

