# Supplementary material for: Cross-sectional and longitudinal associations between active commuting and patterns of movement behaviour during discretionary time: A compositional data analysis
Source: PLoS One. 2019 Aug 16;14(8):e0216650. doi: 10.1371/journal.pone.0216650 (PMC6697339; doi:10.1371/journal.pone.0216650)
Supplement: S1 Table — (DOCX) [file pone.0216650.s001.docx]

S1 Table: Baseline characteristics of the cross-sectional sample and the baseline sample not included in analysis

| **Variable** | **Cross-sectional sample**  (n=182,406) | **Baseline sample not included in analysis**  (n=320,211) |
| --- | --- | --- |
|  | Mean (SD) or n (%) | Mean (SD) or n (%) |
| Age (years) | 52.2 (6.9) | 59.0 (7.7) |
| Sex |  |  |
| Male | 89,946 (49.3) | 139,217 (43.5) |
| Female | 92,460 (50.7) | 180,994 (56.5) |
| Ethnicity |  |  |
| White | 173,057 (94.9) | 299,815 (94.4) |
| Mixed | 1,154 (0.6) | 1,805 (0.6) |
| Asian | 3,521 (1.9) | 6,361 (2.0) |
| Black | 2,665 (1.5) | 5,408 (1.7) |
| Chinese | 626 (0.3) | 949 (0.3) |
| Other | 1,383 (0.8) | 3,155 (1.0) |
| Home ownership |  |  |
| Owner-occupier | 171,134 (93.8) | 271,421 (87.1) |
| Other (e.g. rents) | 11,272 (6.2) | 40,308 (12.9) |
| Car ownership |  |  |
| Owns at least one car | 176,426 (96.7) | 277,323 (87.9) |
| Does not own a car | 5,980 (3.3) | 38,244 (12.1) |
| Household income |  |  |
| <£18,000 | 12,179 (6.7) | 85,041 (35.0) |
| £18,000-30,999 | 34,578 (19.0) | 73,619 (30.3) |
| £31,000-51,999 | 59,688 (32.7) | 51,102 (21.0) |
| £52,000-100,000 | 60,268 (33.0) | 26,011 (10.7) |
| >£100,000 | 15,693 (8.6) | 7,240 (3.0) |
| Education level |  |  |
| University or college degree | 71,115 (39.0) | 90,083 (29.1) |
| Further education | 22,987 (12.6) | 32,344 (10.4) |
| Higher secondary education | 39,892 (21.9) | 65,326 (21.1) |
| Secondary education | 12,918 (7.1) | 13,975 (4.5) |
| Vocational qualifications | 12,378 (6.8) | 20,356 (6.6) |
| Other professional qualifications | 7,624 (4.2) | 18,186 (5.9) |
| None of the above | 15,492 (8.5) | 69,799 (22.5) |
| Has at least one child |  |  |
| Yes | 175,466 (96.2) | 211,095 (94.7) |
| No | 6,940 (3.8) | 11,770 (5.3) |
| Townsend score^a^ | -1.7 (2.8) | -1.1 (3.2) |
| Body mass index (kg/m^2^) | 27.2 (4.6) | 27.6 (4.9) |
| Bone fracture in the preceding five years |  |  |
| Yes | 15,744 (8.6) | 31,727 (10.0) |
| No | 166,662 (91.4) | 284,678 (90.0) |
| Non-vascular condition or disability^b^ |  |  |
| Yes | 38,330 (21.0) | 104,904 (32.9) |
| No | 144,076 (79.0) | 214,001 (67.1) |
| Vascular condition^c^ |  |  |
| Yes | 39,542 (21.7) | 109,807 (34.5) |
| No | 142,864 (78.3) | 208,180 (65.5) |

kg – kilogram; m – metre; n – number; SD – standard deviation

^a^Range from -6.3 to 11.0, where higher scores indicate higher levels of deprivation

^b^Defined according to whether participants reported ever receiving a doctor’s diagnosis for diabetes, cancer or 'any other serious medical conditions or disabilities'

^c^Defined according to whether participants reported ever receiving a doctor’s diagnosis for angina, heart attack, high blood pressure or stroke

S2 Table: Sensitivity analysis for cross-sectional association between commute mode and screen time, walking for pleasure, sport/DIY and total discretionary time (n=237,036)

| **Part** | **Beta coefficient (95% CI)** | | |
| --- | --- | --- | --- |
|  | *Model 1* | *Model 2* | *Model 3* |
| Screen time : rest^a^ | -0.09  (-0.10 to -0.07) | -0.17 (-0.18 to -0.15) | -0.12  (-0.14 to – 0.11) |
| Walking for pleasure : rest^a^ | 0.17  (0.15 to 0.18) | 0.13  (0.11 to 0.14) | 0.11  (0.09 to 0.13) |
| Sport and DIY activities : rest^a^ | -0.08  (-0.10 to -0.06) | 0.04 (0.02 to 0.06) | 0.01 (0.00 to 0.03) |
| Total discretionary time | -0.06  (-0.07 to -0.06) | -0.05  (-0.05 to -0.05) | -0.04  (-0.04 to -0.03) |

CI – confidence interval; DIY - do-it-yourself

^a^Coefficients are for active travel mode with inactive travel mode as the reference category. A positive coefficient indicates that those who used active modes of travel engaged in more of that part relative to the other activities, and a negative coefficient indicates that those who used active modes of travel engaged in less of that part relative to the other activities

Model 1 is unadjusted

Model 2 is adjusted for weekly frequency of travel, distance in miles between home and work, age, sex, ethnicity, home ownership, car ownership, education level and Townsend score

Model 3 is adjusted for the covariates in Model 2 plus body mass index, whether job entailed standing, walking or manual labour, bone fracture in the last five years, ever being diagnosed with a vascular condition and ever being diagnosed with a non-vascular condition

S3 Table: Sensitivity analysis for longitudinal association between commute mode and screen time, walking for pleasure, sport/DIY and total discretionary time (n=5,967)

| **Part** | **Beta coefficient (95% CI)** | | |
| --- | --- | --- | --- |
|  | *Model 1* | *Model 2* | *Model 3* |
| *Screen time : rest*^a^ |  |  |  |
| stable inactive | ref | ref | ref |
| stable active | -0.14  (-0.21 to -0.06) | -0.18  (-0.25 to -0.10) | -0.15  (-0.23 to -0.08) |
| inactive to active | -0.02  (-0.13 to 0.09) | -0.03  (-0.14 to 0.08) | -0.02  (-0.13 to 0.09) |
| active to inactive | 0.02  (-0.10 to 0.14) | 0.05  (-0.11 to 0.12) | 0.01  (-0.11 to 0.13) |
| *Walking for pleasure : rest*^a^ |  |  |  |
| stable inactive | ref | ref | ref |
| stable active | 0.17 (0.06 to 0.28) | 0.15 (0.03 to 0.26) | 0.14  (0.02 to 0.25) |
| inactive to active | -0.02  (-0.18 to 0.14) | -0.03  (-0.20 to 0.13) | -0.04  (-0.20 to 0.13) |
| active to inactive | -0.01  (-0.19 to 0.16) | -0.01  (-0.19 to 0.17) | -0.02 (-0.20 to 0.16) |
| *Sport and DIY activities : rest*^a^ |  |  |  |
| stable inactive | ref | ref | ref |
| stable active | 0.00  (-0.12 to 0.10) | 0.06  (-0.05 to 0.17) | 0.04  (-0.07 to 0.15) |
| inactive to active | 0.09  (-0.07 to 0.24) | 0.10  (-0.06 to 0.26) | 0.09  (-0.06 to 0.25) |
| active to inactive | 0.02  (-0.15 to 0.19) | 0.03  (-0.14 to 0.20) | 0.03  (-0.14 to 0.20) |
| *Total discretionary time* |  |  |  |
| stable inactive | ref | ref | ref |
| stable active | -0.05  (-0.07 to -0.02) | -0.05  (-0.07 to -0.02) | -0.03  (-0.06 to -0.01) |
| inactive to active | 0.03  (-0.01 to 0.06) | 0.03  (-0.01 to 0.07) | 0.03  (-0.01 to 0.07) |
| active to inactive | -0.01  (-0.05 to 0.03) | -0.01  (-0.05 to 0.03) | -0.01  (-0.05 to 0.03) |

CI – confidence interval; DIY - do-it-yourself

^a^Coefficients are for a particular commute category with stable inactive as the reference category. A positive coefficient indicates that those in a particular commute category engaged in more of that part relative to the other activities, and a negative coefficient indicates that those in a particular commute category travel engaged in less of that part relative to the other activities

Model 1 is unadjusted

Model 2 is adjusted for weekly frequency of travel, distance in miles between home and work, age, sex, ethnicity, home ownership, car ownership, education level and Townsend score

Model 3 is adjusted for the covariates in Model 2 plus body mass index, whether job entailed standing, walking or manual labour, bone fracture in the last five years, ever being diagnosed with a vascular condition, ever being diagnosed with a non-vascular condition, time elapsed between assessments and whether the season differed between assessments
