# Supplementary material for: Cross-sectional and longitudinal associations between active commuting and patterns of movement behaviour during discretionary time: A compositional data analysis
Source: PLoS One. 2019 Aug 16;14(8):e0216650. doi: 10.1371/journal.pone.0216650 (PMC6697339; doi:10.1371/journal.pone.0216650)
Supplement: S2 Table — (DOCX) [file pone.0216650.s002.docx]

S2 Table: Sensitivity analysis for cross-sectional association between commute mode and screen time, walking for pleasure, sport/DIY and total discretionary time (n=237,036)

| **Part** | **Beta coefficient (95% CI)** | | |
| --- | --- | --- | --- |
|  | *Model 1* | *Model 2* | *Model 3* |
| Screen time : rest^a^ | -0.09  (-0.10 to -0.07) | -0.17 (-0.18 to -0.15) | -0.12  (-0.14 to – 0.11) |
| Walking for pleasure : rest^a^ | 0.17  (0.15 to 0.18) | 0.13  (0.11 to 0.14) | 0.11  (0.09 to 0.13) |
| Sport and DIY activities : rest^a^ | -0.08  (-0.10 to -0.06) | 0.04 (0.02 to 0.06) | 0.01 (0.00 to 0.03) |
| Total discretionary time | -0.06  (-0.07 to -0.06) | -0.05  (-0.05 to -0.05) | -0.04  (-0.04 to -0.03) |

CI – confidence interval; DIY - do-it-yourself

^a^Coefficients are for active travel mode with inactive travel mode as the reference category. A positive coefficient indicates that those who used active modes of travel engaged in more of that part relative to the other activities, and a negative coefficient indicates that those who used active modes of travel engaged in less of that part relative to the other activities

Model 1 is unadjusted

Model 2 is adjusted for weekly frequency of travel, distance in miles between home and work, age, sex, ethnicity, home ownership, car ownership, education level and Townsend score

Model 3 is adjusted for the covariates in Model 2 plus body mass index, whether job entailed standing, walking or manual labour, bone fracture in the last five years, ever being diagnosed with a vascular condition and ever being diagnosed with a non-vascular condition
