# Supplementary material for: Cross-sectional and longitudinal associations between active commuting and patterns of movement behaviour during discretionary time: A compositional data analysis
Source: PLoS One. 2019 Aug 16;14(8):e0216650. doi: 10.1371/journal.pone.0216650 (PMC6697339; doi:10.1371/journal.pone.0216650)
Supplement: S3 Table — (DOCX) [file pone.0216650.s003.docx]

S3 Table: Sensitivity analysis for longitudinal association between commute mode and screen time, walking for pleasure, sport/DIY and total discretionary time (n=5,967)

| **Part** | **Beta coefficient (95% CI)** | | |
| --- | --- | --- | --- |
|  | *Model 1* | *Model 2* | *Model 3* |
| *Screen time : rest*^a^ |  |  |  |
| stable inactive | ref | ref | ref |
| stable active | -0.14  (-0.21 to -0.06) | -0.18  (-0.25 to -0.10) | -0.15  (-0.23 to -0.08) |
| inactive to active | -0.02  (-0.13 to 0.09) | -0.03  (-0.14 to 0.08) | -0.02  (-0.13 to 0.09) |
| active to inactive | 0.02  (-0.10 to 0.14) | 0.05  (-0.11 to 0.12) | 0.01  (-0.11 to 0.13) |
| *Walking for pleasure : rest*^a^ |  |  |  |
| stable inactive | ref | ref | ref |
| stable active | 0.17 (0.06 to 0.28) | 0.15 (0.03 to 0.26) | 0.14  (0.02 to 0.25) |
| inactive to active | -0.02  (-0.18 to 0.14) | -0.03  (-0.20 to 0.13) | -0.04  (-0.20 to 0.13) |
| active to inactive | -0.01  (-0.19 to 0.16) | -0.01  (-0.19 to 0.17) | -0.02 (-0.20 to 0.16) |
| *Sport and DIY activities : rest*^a^ |  |  |  |
| stable inactive | ref | ref | ref |
| stable active | 0.00  (-0.12 to 0.10) | 0.06  (-0.05 to 0.17) | 0.04  (-0.07 to 0.15) |
| inactive to active | 0.09  (-0.07 to 0.24) | 0.10  (-0.06 to 0.26) | 0.09  (-0.06 to 0.25) |
| active to inactive | 0.02  (-0.15 to 0.19) | 0.03  (-0.14 to 0.20) | 0.03  (-0.14 to 0.20) |
| *Total discretionary time* |  |  |  |
| stable inactive | ref | ref | ref |
| stable active | -0.05  (-0.07 to -0.02) | -0.05  (-0.07 to -0.02) | -0.03  (-0.06 to -0.01) |
| inactive to active | 0.03  (-0.01 to 0.06) | 0.03  (-0.01 to 0.07) | 0.03  (-0.01 to 0.07) |
| active to inactive | -0.01  (-0.05 to 0.03) | -0.01  (-0.05 to 0.03) | -0.01  (-0.05 to 0.03) |

CI – confidence interval; DIY - do-it-yourself

^a^Coefficients are for a particular commute category with stable inactive as the reference category. A positive coefficient indicates that those in a particular commute category engaged in more of that part relative to the other activities, and a negative coefficient indicates that those in a particular commute category travel engaged in less of that part relative to the other activities

Model 1 is unadjusted

Model 2 is adjusted for weekly frequency of travel, distance in miles between home and work, age, sex, ethnicity, home ownership, car ownership, education level and Townsend score

Model 3 is adjusted for the covariates in Model 2 plus body mass index, whether job entailed standing, walking or manual labour, bone fracture in the last five years, ever being diagnosed with a vascular condition, ever being diagnosed with a non-vascular condition, time elapsed between assessments and whether the season differed between assessments
