# Supplementary material for: Cross-sectional and longitudinal associations between active commuting and patterns of movement behaviour during discretionary time: A compositional data analysis
Source: PLoS One. 2019 Aug 16;14(8):e0216650. doi: 10.1371/journal.pone.0216650 (PMC6697339; doi:10.1371/journal.pone.0216650)
Supplement: S4 Table — (DOCX) [file pone.0216650.s004.docx]

S4 Table: Biobank variables comprising core analysis dataset

| **Variable name** | **Variable description** |
| --- | --- |
| n_eid | Encoded anonymised participant ID |
| n_31_0_0 | Sex |
| n_34_0_0 | Year of birth |
| n_52_0_0 | Month of birth |
| n_54_0_0 | UK Biobank assessment centre |
| n_54_1_0 | UK Biobank assessment centre |
| n_54_2_0 | UK Biobank assessment centre |
| n_130_0_0 | Place of birth in UK - east co-ordinate |
| n_130_1_0 | Place of birth in UK - east co-ordinate |
| n_130_2_0 | Place of birth in UK - east co-ordinate |
| n_132_0_0 | Job code |
| n_132_1_0 | Job code |
| n_132_2_0 | Job code |
| n_189_0_0 | Townsend deprivation index at recruitment |
| n_680_0_0 | Own or rent accommodation lived in |
| n_680_1_0 | Own or rent accommodation lived in |
| n_680_2_0 | Own or rent accommodation lived in |
| n_699_0_0 | Length of time at current address |
| n_699_1_0 | Length of time at current address |
| n_699_2_0 | Length of time at current address |
| n_709_0_0 | Number in household |
| n_709_1_0 | Number in household |
| n_709_2_0 | Number in household |
| n_728_0_0 | Number of vehicles in household |
| n_728_1_0 | Number of vehicles in household |
| n_728_2_0 | Number of vehicles in household |
| n_738_0_0 | Average total household income before tax |
| n_738_1_0 | Average total household income before tax |
| n_738_2_0 | Average total household income before tax |
| n_757_0_0 | Time employed in main current job |
| n_757_1_0 | Time employed in main current job |
| n_757_2_0 | Time employed in main current job |
| n_767_0_0 | Length of working week for main job |
| n_767_1_0 | Length of working week for main job |
| n_767_2_0 | Length of working week for main job |
| n_826_0_0 | Job involves shift work |
| n_826_1_0 | Job involves shift work |
| n_826_2_0 | Job involves shift work |
| n_845_0_0 | Age completed full time education |
| n_845_1_0 | Age completed full time education |
| n_845_2_0 | Age completed full time education |
| n_1070_0_0 | Time spent watching television (TV) |
| n_1070_1_0 | Time spent watching television (TV) |
| n_1070_2_0 | Time spent watching television (TV) |
| n_3140_0_0 | Pregnant |
| n_3140_1_0 | Pregnant |
| n_3140_2_0 | Pregnant |
| n_3426_0_0 | Job involve night shift work |
| n_3426_1_0 | Job involve night shift work |
| n_3426_2_0 | Job involve night shift work |
| n_6138_0_0 | Qualifications |
| n_6138_0_1 | Qualifications |
| n_6138_0_2 | Qualifications |
| n_6138_0_3 | Qualifications |
| n_6138_0_4 | Qualifications |
| n_6138_0_5 | Qualifications |
| n_6138_1_0 | Qualifications |
| n_6138_1_1 | Qualifications |
| n_6138_1_2 | Qualifications |
| n_6138_1_3 | Qualifications |
| n_6138_1_4 | Qualifications |
| n_6138_1_5 | Qualifications |
| n_6138_2_0 | Qualifications |
| n_6138_2_1 | Qualifications |
| n_6138_2_2 | Qualifications |
| n_6138_2_3 | Qualifications |
| n_6138_2_4 | Qualifications |
| n_6138_2_5 | Qualifications |
| n_6141_0_0 | How are people in household related to participant |
| n_6141_0_1 | How are people in household related to participant |
| n_6141_0_2 | How are people in household related to participant |
| n_6141_0_3 | How are people in household related to participant |
| n_6141_0_4 | How are people in household related to participant |
| n_6141_1_0 | How are people in household related to participant |
| n_6141_1_1 | How are people in household related to participant |
| n_6141_1_2 | How are people in household related to participant |
| n_6141_1_3 | How are people in household related to participant |
| n_6141_1_4 | How are people in household related to participant |
| n_6141_2_0 | How are people in household related to participant |
| n_6141_2_1 | How are people in household related to participant |
| n_6141_2_2 | How are people in household related to participant |
| n_6141_2_3 | How are people in household related to participant |
| n_6141_2_4 | How are people in household related to participant |
| n_6142_0_0 | Current employment status |
| n_6142_0_1 | Current employment status |
| n_6142_0_2 | Current employment status |
| n_6142_0_3 | Current employment status |
| n_6142_0_4 | Current employment status |
| n_6142_0_5 | Current employment status |
| n_6142_0_6 | Current employment status |
| n_6142_1_0 | Current employment status |
| n_6142_1_1 | Current employment status |
| n_6142_1_2 | Current employment status |
| n_6142_1_3 | Current employment status |
| n_6142_1_4 | Current employment status |
| n_6142_2_0 | Current employment status |
| n_6142_2_1 | Current employment status |
| n_6142_2_2 | Current employment status |
| n_6142_2_3 | Current employment status |
| n_20024_0_0 | Job code - deduced |
| n_20074_0_0 | Home location at assessment - east co-ordinate (rounded) |
| n_20074_1_0 | Home location at assessment - east co-ordinate (rounded) |
| n_20075_0_0 | Home location at assessment - north co-ordinate (rounded) |
| n_20075_1_0 | Home location at assessment - north co-ordinate (rounded) |
| n_20119_0_0 | Current employment status - corrected |
| n_21000_0_0 | Ethnic background |
| n_21000_1_0 | Ethnic background |
| n_21000_2_0 | Ethnic background |
| n_21001_0_0 | Body mass index (BMI) |
| n_21001_1_0 | Body mass index (BMI) |
| n_21001_2_0 | Body mass index (BMI) |
| n_21002_0_0 | Weight |
| n_21002_1_0 | Weight |
| n_21002_2_0 | Weight |
| n_21003_0_0 | Age when attended assessment centre |
| n_21003_1_0 | Age when attended assessment centre |
| n_21003_2_0 | Age when attended assessment centre |
| n_21022_0_0 | Age at recruitment |
| n_23099_0_0 | Body fat percentage |
| n_23099_1_0 | Body fat percentage |
| ts_53_0_0 | Date of attending assessment centre |
| ts_53_1_0 | Date of attending assessment centre |
| ts_53_2_0 | Date of attending assessment centre |
| occ_grp_0_0 | Standard Occupational Classification 2000 (SOC2000) |
| occ_grp_1_0 | Standard Occupational Classification 2000 (SOC2000) |
| occ_grp_2_0 | Standard Occupational Classification 2000 (SOC2000) |
| ethnicity_0_0 | Derived ethnic groups |
| age_0_0 | Derived age (years); based on date of assessment |
| age_1_0 | Derived age (years); based on date of assessment |
| age_2_0 | Derived age (years); based on date of assessment |
| agesquared_0_0 | Derived age-squared (years) |
| agesquared_1_0 | Derived age-squared (years) |
| agesquared_2_0 | Derived age-squared (years) |
| n_23100_0_0 | Whole body fat mass |
| n_23100_1_0 | Whole body fat mass |
| n_23101_0_0 | Whole body fat-free mass |
| n_23101_1_0 | Whole body fat-free mass |
| n_23127_0_0 | Trunk fat percentage |
| n_23127_1_0 | Trunk fat percentage |
| ts_110008_1_0 | Invitation to assessment centre, date sent |
| ts_110008_2_0 | Invitation to assessment centre, date sent |
| n_864_0_0 | Number of days/week walked 10+ minutes |
| n_864_1_0 | Number of days/week walked 10+ minutes |
| n_864_2_0 | Number of days/week walked 10+ minutes |
| n_874_0_0 | Duration of walks |
| n_874_1_0 | Duration of walks |
| n_874_2_0 | Duration of walks |
| n_884_0_0 | Number of days/week of moderate physical activity 10+ minutes |
| n_884_1_0 | Number of days/week of moderate physical activity 10+ minutes |
| n_884_2_0 | Number of days/week of moderate physical activity 10+ minutes |
| n_894_0_0 | Duration of moderate activity |
| n_894_1_0 | Duration of moderate activity |
| n_894_2_0 | Duration of moderate activity |
| n_904_0_0 | Number of days/week of vigorous physical activity 10+ minutes |
| n_904_1_0 | Number of days/week of vigorous physical activity 10+ minutes |
| n_904_2_0 | Number of days/week of vigorous physical activity 10+ minutes |
| n_914_0_0 | Duration of vigorous activity |
| n_914_1_0 | Duration of vigorous activity |
| n_914_2_0 | Duration of vigorous activity |
| n_924_0_0 | Usual walking pace |
| n_924_1_0 | Usual walking pace |
| n_924_2_0 | Usual walking pace |
| n_943_0_0 | Frequency of stair climbing in last 4 weeks |
| n_943_1_0 | Frequency of stair climbing in last 4 weeks |
| n_943_2_0 | Frequency of stair climbing in last 4 weeks |
| n_971_0_0 | Frequency of walking for pleasure in last 4 weeks |
| n_971_1_0 | Frequency of walking for pleasure in last 4 weeks |
| n_971_2_0 | Frequency of walking for pleasure in last 4 weeks |
| n_981_0_0 | Duration walking for pleasure |
| n_981_1_0 | Duration walking for pleasure |
| n_981_2_0 | Duration walking for pleasure |
| n_991_0_0 | Frequency of strenuous sports in last 4 weeks |
| n_991_1_0 | Frequency of strenuous sports in last 4 weeks |
| n_991_2_0 | Frequency of strenuous sports in last 4 weeks |
| n_1001_0_0 | Duration of strenuous sports |
| n_1001_1_0 | Duration of strenuous sports |
| n_1001_2_0 | Duration of strenuous sports |
| n_1011_0_0 | Frequency of light DIY in last 4 weeks |
| n_1011_1_0 | Frequency of light DIY in last 4 weeks |
| n_1011_2_0 | Frequency of light DIY in last 4 weeks |
| n_1021_0_0 | Duration of light DIY |
| n_1021_1_0 | Duration of light DIY |
| n_1021_2_0 | Duration of light DIY |
| n_1031_0_0 | Frequency of friend/family visits |
| n_1031_1_0 | Frequency of friend/family visits |
| n_1031_2_0 | Frequency of friend/family visits |
| n_1080_0_0 | Time spent using computer |
| n_1080_1_0 | Time spent using computer |
| n_1080_2_0 | Time spent using computer |
| n_1090_0_0 | Time spent driving |
| n_1090_1_0 | Time spent driving |
| n_1090_2_0 | Time spent driving |
| n_1100_0_0 | Drive faster than motorway speed limit |
| n_1100_1_0 | Drive faster than motorway speed limit |
| n_1100_2_0 | Drive faster than motorway speed limit |
| n_1160_0_0 | Sleep duration |
| n_1160_1_0 | Sleep duration |
| n_1160_2_0 | Sleep duration |
| n_1170_0_0 | Getting up in morning |
| n_1170_1_0 | Getting up in morning |
| n_1170_2_0 | Getting up in morning |
| n_1180_0_0 | Morning/evening person (chronotype) |
| n_1180_1_0 | Morning/evening person (chronotype) |
| n_1180_2_0 | Morning/evening person (chronotype) |
| n_1190_0_0 | Nap during day |
| n_1190_1_0 | Nap during day |
| n_1190_2_0 | Nap during day |
| n_1200_0_0 | Sleeplessness / insomnia |
| n_1200_1_0 | Sleeplessness / insomnia |
| n_1200_2_0 | Sleeplessness / insomnia |
| n_1220_0_0 | Daytime dozing / sleeping (narcolepsy) |
| n_1220_1_0 | Daytime dozing / sleeping (narcolepsy) |
| n_1220_2_0 | Daytime dozing / sleeping (narcolepsy) |
| n_2624_0_0 | Frequency of heavy DIY in last 4 weeks |
| n_2624_1_0 | Frequency of heavy DIY in last 4 weeks |
| n_2624_2_0 | Frequency of heavy DIY in last 4 weeks |
| n_2634_0_0 | Duration of heavy DIY |
| n_2634_1_0 | Duration of heavy DIY |
| n_2634_2_0 | Duration of heavy DIY |
| n_3637_0_0 | Frequency of other exercises in last 4 weeks |
| n_3637_1_0 | Frequency of other exercises in last 4 weeks |
| n_3637_2_0 | Frequency of other exercises in last 4 weeks |
| n_3647_0_0 | Duration of other exercises |
| n_3647_1_0 | Duration of other exercises |
| n_3647_2_0 | Duration of other exercises |
| n_6162_0_0 | Types of transport used (excluding work) |
| n_6162_0_1 | Types of transport used (excluding work) |
| n_6162_0_2 | Types of transport used (excluding work) |
| n_6162_0_3 | Types of transport used (excluding work) |
| n_6162_1_0 | Types of transport used (excluding work) |
| n_6162_1_1 | Types of transport used (excluding work) |
| n_6162_1_2 | Types of transport used (excluding work) |
| n_6162_1_3 | Types of transport used (excluding work) |
| n_6162_2_0 | Types of transport used (excluding work) |
| n_6162_2_1 | Types of transport used (excluding work) |
| n_6162_2_2 | Types of transport used (excluding work) |
| n_6162_2_3 | Types of transport used (excluding work) |
| n_6164_0_0 | Types of physical activity in last 4 weeks |
| n_6164_0_1 | Types of physical activity in last 4 weeks |
| n_6164_0_2 | Types of physical activity in last 4 weeks |
| n_6164_0_3 | Types of physical activity in last 4 weeks |
| n_6164_0_4 | Types of physical activity in last 4 weeks |
| n_6164_1_0 | Types of physical activity in last 4 weeks |
| n_6164_1_1 | Types of physical activity in last 4 weeks |
| n_6164_1_2 | Types of physical activity in last 4 weeks |
| n_6164_1_3 | Types of physical activity in last 4 weeks |
| n_6164_1_4 | Types of physical activity in last 4 weeks |
| n_6164_2_0 | Types of physical activity in last 4 weeks |
| n_6164_2_1 | Types of physical activity in last 4 weeks |
| n_6164_2_2 | Types of physical activity in last 4 weeks |
| n_6164_2_3 | Types of physical activity in last 4 weeks |
| n_6164_2_4 | Types of physical activity in last 4 weeks |
| n_10953_0_0 | Duration of walks (pilot) |
| n_10962_0_0 | Duration of moderate physical activity (pilot) |
| n_10971_0_0 | Duration of vigorous physical activity (pilot) |
| n_777_0_0 | Frequency of travelling from home to job workplace |
| n_777_1_0 | Frequency of travelling from home to job workplace |
| n_777_2_0 | Frequency of travelling from home to job workplace |
| n_796_0_0 | Distance between home and job workplace |
| n_796_1_0 | Distance between home and job workplace |
| n_796_2_0 | Distance between home and job workplace |
| n_806_0_0 | Job involves mainly walking or standing |
| n_806_1_0 | Job involves mainly walking or standing |
| n_806_2_0 | Job involves mainly walking or standing |
| n_816_0_0 | Job involves heavy manual or physical work |
| n_816_1_0 | Job involves heavy manual or physical work |
| n_816_2_0 | Job involves heavy manual or physical work |
| n_6143_0_0 | Transport type for commuting to job workplace |
| n_6143_0_1 | Transport type for commuting to job workplace |
| n_6143_0_2 | Transport type for commuting to job workplace |
| n_6143_0_3 | Transport type for commuting to job workplace |
| n_6143_1_0 | Transport type for commuting to job workplace |
| n_6143_1_1 | Transport type for commuting to job workplace |
| n_6143_1_2 | Transport type for commuting to job workplace |
| n_6143_1_3 | Transport type for commuting to job workplace |
| n_6143_2_0 | Transport type for commuting to job workplace |
| n_6143_2_1 | Transport type for commuting to job workplace |
| n_6143_2_2 | Transport type for commuting to job workplace |
| n_6143_2_3 | Transport type for commuting to job workplace |
| n_90002__1_0 | Data problem indicator |
| n_90002_0_0 | Data problem indicator |
| n_90002_1_0 | Data problem indicator |
| n_90012_0_0 | Overall acceleration average |
| n_90013_0_0 | Standard deviation of acceleration |
| n_90015_0_0 | Data quality, good wear time |
| n_90016_0_0 | Data quality, good calibration |
| n_90017_0_0 | Data quality, calibrated on own data |
| n_90018_0_0 | Daylight savings crossover |
| n_90019_0_0 | Monday average acceleration |
| n_90020_0_0 | Tuesday average acceleration |
| n_90021_0_0 | Wednesday average acceleration |
| n_90022_0_0 | Thursday average acceleration |
| n_90023_0_0 | Friday average acceleration |
| n_90024_0_0 | Saturday average acceleration |
| n_90025_0_0 | Sunday average acceleration |
| n_90026_0_0 | First weekday of wear |
| n_90027_0_0 | Average acceleration 00:00 - 00:59 |
| n_90028_0_0 | Average acceleration 01:00 - 01:59 |
| n_90029_0_0 | Average acceleration 02:00 - 02:59 |
| n_90030_0_0 | Average acceleration 03:00 - 03:59 |
| n_90031_0_0 | Average acceleration 04:00 - 04:59 |
| n_90032_0_0 | Average acceleration 05:00 - 05:59 |
| n_90033_0_0 | Average acceleration 06:00 - 06:59 |
| n_90034_0_0 | Average acceleration 07:00 - 07:59 |
| n_90035_0_0 | Average acceleration 08:00 - 08:59 |
| n_90036_0_0 | Average acceleration 09:00 - 09:59 |
| n_90037_0_0 | Average acceleration 10:00 - 10:59 |
| n_90038_0_0 | Average acceleration 11:00 - 11:59 |
| n_90039_0_0 | Average acceleration 12:00 - 12:59 |
| n_90040_0_0 | Average acceleration 13:00 - 13:59 |
| n_90041_0_0 | Average acceleration 14:00 - 14:59 |
| n_90042_0_0 | Average acceleration 15:00 - 15:59 |
| n_90043_0_0 | Average acceleration 16:00 - 16:59 |
| n_90044_0_0 | Average acceleration 17:00 - 17:59 |
| n_90045_0_0 | Average acceleration 18:00 - 18:59 |
| n_90046_0_0 | Average acceleration 19:00 - 19:59 |
| n_90047_0_0 | Average acceleration 20:00 - 20:59 |
| n_90048_0_0 | Average acceleration 21:00 - 21:59 |
| n_90049_0_0 | Average acceleration 22:00 - 22:59 |
| n_90050_0_0 | Average acceleration 23:00 - 23:59 |
| n_90051_0_0 | Wear duration overall |
| n_90052_0_0 | Non wear duration overall |
| n_90053_0_0 | Wear duration during Monday |
| n_90054_0_0 | Wear duration during Tuesday |
| n_90055_0_0 | Wear duration during Wednesday |
| n_90056_0_0 | Wear duration during Thursday |
| n_90057_0_0 | Wear duration during Friday |
| n_90058_0_0 | Wear duration during Saturday |
| n_90059_0_0 | Wear duration during Sunday |
| n_90060_0_0 | Wear duration during 00:00 - 00:59 |
| n_90061_0_0 | Wear duration during 01:00 - 01:59 |
| n_90062_0_0 | Wear duration during 02:00 - 02:59 |
| n_90063_0_0 | Wear duration during 03:00 - 03:59 |
| n_90064_0_0 | Wear duration during 04:00 - 04:59 |
| n_90065_0_0 | Wear duration during 05:00 - 05:59 |
| n_90066_0_0 | Wear duration during 06:00 - 06:59 |
| n_90067_0_0 | Wear duration during 07:00 - 07:59 |
| n_90068_0_0 | Wear duration during 08:00 - 08:59 |
| n_90069_0_0 | Wear duration during 09:00 - 09:59 |
| n_90070_0_0 | Wear duration during 10:00 - 10:59 |
| n_90071_0_0 | Wear duration during 11:00 - 11:59 |
| n_90072_0_0 | Wear duration during 12:00 - 12:59 |
| n_90073_0_0 | Wear duration during 13:00 - 13:59 |
| n_90074_0_0 | Wear duration during 14:00 - 14:59 |
| n_90075_0_0 | Wear duration during 15:00 - 15:59 |
| n_90076_0_0 | Wear duration during 16:00 - 16:59 |
| n_90077_0_0 | Wear duration during 17:00 - 17:59 |
| n_90078_0_0 | Wear duration during 18:00 - 18:59 |
| n_90079_0_0 | Wear duration during 19:00 - 19:59 |
| n_90080_0_0 | Wear duration during 20:00 - 20:59 |
| n_90081_0_0 | Wear duration during 21:00 - 21:59 |
| n_90082_0_0 | Wear duration during 22:00 - 22:59 |
| n_90083_0_0 | Wear duration during 23:00 - 23:59 |
| n_90084_0_0 | Unique hours of wear in a 24 hour cycle (scattered over multiple days) |
| n_90085_0_0 | Unique minutes of wear in a 24 hour cycle (scattered over multiple days) |
| n_90086_0_0 | Non wear episodes over 1 hour duration |
| n_90087_0_0 | No-wear time bias adjusted average acceleration |
| n_90088_0_0 | No-wear time bias adjusted acceleration standard deviation |
| n_90089_0_0 | No-wear time bias adjusted acceleration median |
| n_90090_0_0 | No-wear time bias adjusted acceleration minimum |
| n_90091_0_0 | No-wear time bias adjusted acceleration maximum |
| n_90092_0_0 | Fraction acceleration <= 1 milli-gravities |
| n_90093_0_0 | Fraction acceleration <= 2 milli-gravities |
| n_90094_0_0 | Fraction acceleration <= 3 milli-gravities |
| n_90095_0_0 | Fraction acceleration <= 4 milli-gravities |
| n_90096_0_0 | Fraction acceleration <= 5 milli-gravities |
| n_90097_0_0 | Fraction acceleration <= 6 milli-gravities |
| n_90098_0_0 | Fraction acceleration <= 7 milli-gravities |
| n_90099_0_0 | Fraction acceleration <= 8 milli-gravities |
| n_90100_0_0 | Fraction acceleration <= 9 milli-gravities |
| n_90101_0_0 | Fraction acceleration <= 10 milli-gravities |
| n_90102_0_0 | Fraction acceleration <= 11 milli-gravities |
| n_90103_0_0 | Fraction acceleration <= 12 milli-gravities |
| n_90104_0_0 | Fraction acceleration <= 13 milli-gravities |
| n_90105_0_0 | Fraction acceleration <= 14 milli-gravities |
| n_90106_0_0 | Fraction acceleration <= 15 milli-gravities |
| n_90107_0_0 | Fraction acceleration <= 16 milli-gravities |
| n_90108_0_0 | Fraction acceleration <= 17 milli-gravities |
| n_90109_0_0 | Fraction acceleration <= 18 milli-gravities |
| n_90110_0_0 | Fraction acceleration <= 19 milli-gravities |
| n_90111_0_0 | Fraction acceleration <= 20 milli-gravities |
| n_90112_0_0 | Fraction acceleration <= 25 milli-gravities |
| n_90113_0_0 | Fraction acceleration <= 30 milli-gravities |
| n_90114_0_0 | Fraction acceleration <= 35 milli-gravities |
| n_90115_0_0 | Fraction acceleration <= 40 milli-gravities |
| n_90116_0_0 | Fraction acceleration <= 45 milli-gravities |
| n_90117_0_0 | Fraction acceleration <= 50 milli-gravities |
| n_90118_0_0 | Fraction acceleration <= 55 milli-gravities |
| n_90119_0_0 | Fraction acceleration <= 60 milli-gravities |
| n_90120_0_0 | Fraction acceleration <= 65 milli-gravities |
| n_90121_0_0 | Fraction acceleration <= 70 milli-gravities |
| n_90122_0_0 | Fraction acceleration <= 75 milli-gravities |
| n_90123_0_0 | Fraction acceleration <= 80 milli-gravities |
| n_90124_0_0 | Fraction acceleration <= 85 milli-gravities |
| n_90125_0_0 | Fraction acceleration <= 90 milli-gravities |
| n_90126_0_0 | Fraction acceleration <= 95 milli-gravities |
| n_90127_0_0 | Fraction acceleration <= 100 milli-gravities |
| n_90128_0_0 | Fraction acceleration <= 125 milli-gravities |
| n_90129_0_0 | Fraction acceleration <= 150 milli-gravities |
| n_90130_0_0 | Fraction acceleration <= 175 milli-gravities |
| n_90131_0_0 | Fraction acceleration <= 200 milli-gravities |
| n_90132_0_0 | Fraction acceleration <= 225 milli-gravities |
| n_90133_0_0 | Fraction acceleration <= 250 milli-gravities |
| n_90134_0_0 | Fraction acceleration <= 275 milli-gravities |
| n_90135_0_0 | Fraction acceleration <= 300 milli-gravities |
| n_90136_0_0 | Fraction acceleration <= 325 milli-gravities |
| n_90137_0_0 | Fraction acceleration <= 350 milli-gravities |
| n_90138_0_0 | Fraction acceleration <= 375 milli-gravities |
| n_90139_0_0 | Fraction acceleration <= 400 milli-gravities |
| n_90140_0_0 | Fraction acceleration <= 425 milli-gravities |
| n_90141_0_0 | Fraction acceleration <= 450 milli-gravities |
| n_90142_0_0 | Fraction acceleration <= 475 milli-gravities |
| n_90143_0_0 | Fraction acceleration <= 500 milli-gravities |
| n_90144_0_0 | Fraction acceleration <= 600 milli-gravities |
| n_90145_0_0 | Fraction acceleration <= 700 milli-gravities |
| n_90146_0_0 | Fraction acceleration <= 800 milli-gravities |
| n_90147_0_0 | Fraction acceleration <= 900 milli-gravities |
| n_90148_0_0 | Fraction acceleration <= 1000 milli-gravities |
| n_90149_0_0 | Fraction acceleration <= 1100 milli-gravities |
| n_90150_0_0 | Fraction acceleration <= 1200 milli-gravities |
| n_90151_0_0 | Fraction acceleration <= 1300 milli-gravities |
| n_90152_0_0 | Fraction acceleration <= 1400 milli-gravities |
| n_90153_0_0 | Fraction acceleration <= 1500 milli-gravities |
| n_90154_0_0 | Fraction acceleration <= 1600 milli-gravities |
| n_90155_0_0 | Fraction acceleration <= 1700 milli-gravities |
| n_90156_0_0 | Fraction acceleration <= 1800 milli-gravities |
| n_90157_0_0 | Fraction acceleration <= 1900 milli-gravities |
| n_90158_0_0 | Fraction acceleration <= 2000 milli-gravities |
| n_90159_0_0 | Error tolerance before calibration |
| n_90160_0_0 | Error tolerance after calibration |
| n_90179_0_0 | Device ID |
| n_90182_0_0 | Data recording errors |
| n_90187_0_0 | Total data readings |
| n_104900_0_0 | Time spent doing vigorous physical activity |
| n_104900_1_0 | Time spent doing vigorous physical activity |
| n_104900_2_0 | Time spent doing vigorous physical activity |
| n_104900_3_0 | Time spent doing vigorous physical activity |
| n_104900_4_0 | Time spent doing vigorous physical activity |
| n_104910_0_0 | Time spent doing moderate physical activity |
| n_104910_1_0 | Time spent doing moderate physical activity |
| n_104910_2_0 | Time spent doing moderate physical activity |
| n_104910_3_0 | Time spent doing moderate physical activity |
| n_104910_4_0 | Time spent doing moderate physical activity |
| n_104920_0_0 | Time spent doing light physical activity |
| n_104920_1_0 | Time spent doing light physical activity |
| n_104920_2_0 | Time spent doing light physical activity |
| n_104920_3_0 | Time spent doing light physical activity |
| n_104920_4_0 | Time spent doing light physical activity |
| n_110005_0_0 | Invitation to physical activity study, acceptance |
| ts_90010_0_0 | Start time of wear |
| ts_90011_0_0 | End time of wear |
| ts_110006_0_0 | Invitation to physical activity study, date sent |
| n_6032_0_0 | Maximum workload during fitness test |
| n_6032_1_0 | Maximum workload during fitness test |
| n_6033_0_0 | Maximum heart rate during fitness test |
| n_6033_1_0 | Maximum heart rate during fitness test |
| n_6034_0_0 | Target heart rate achieved |
| n_6034_1_0 | Target heart rate achieved |
| n_6039_0_0 | Duration of fitness test |
| n_6039_1_0 | Duration of fitness test |
| n_2335_0_0 | Chest pain or discomfort |
| n_2335_1_0 | Chest pain or discomfort |
| n_2335_2_0 | Chest pain or discomfort |
| n_3606_0_0 | Chest pain or discomfort walking normally |
| n_3606_1_0 | Chest pain or discomfort walking normally |
| n_3606_2_0 | Chest pain or discomfort walking normally |
| n_3616_0_0 | Chest pain due to walking ceases when standing still |
| n_3616_1_0 | Chest pain due to walking ceases when standing still |
| n_3616_2_0 | Chest pain due to walking ceases when standing still |
| n_3751_0_0 | Chest pain or discomfort when walking uphill or hurrying |
| n_3751_1_0 | Chest pain or discomfort when walking uphill or hurrying |
| n_3751_2_0 | Chest pain or discomfort when walking uphill or hurrying |
| n_4728_0_0 | Leg pain on walking |
| n_4728_1_0 | Leg pain on walking |
| n_4728_2_0 | Leg pain on walking |
| n_5463_0_0 | Leg pain in calf/calves |
| n_5463_1_0 | Leg pain in calf/calves |
| n_5463_2_0 | Leg pain in calf/calves |
| n_5474_0_0 | Leg pain when walking uphill or hurrying |
| n_5474_1_0 | Leg pain when walking uphill or hurrying |
| n_5474_2_0 | Leg pain when walking uphill or hurrying |
| n_6014_0_0 | Doctor restricts physical activity due to heart condition |
| n_6014_1_0 | Doctor restricts physical activity due to heart condition |
| n_6145_0_0 | Illness, injury, bereavement, stress in last 2 years |
| n_6145_0_1 | Illness, injury, bereavement, stress in last 2 years |
| n_6145_0_2 | Illness, injury, bereavement, stress in last 2 years |
| n_6145_0_3 | Illness, injury, bereavement, stress in last 2 years |
| n_6145_0_4 | Illness, injury, bereavement, stress in last 2 years |
| n_6145_0_5 | Illness, injury, bereavement, stress in last 2 years |
| n_6145_1_0 | Illness, injury, bereavement, stress in last 2 years |
| n_6145_1_1 | Illness, injury, bereavement, stress in last 2 years |
| n_6145_1_2 | Illness, injury, bereavement, stress in last 2 years |
| n_6145_1_3 | Illness, injury, bereavement, stress in last 2 years |
| n_6145_1_4 | Illness, injury, bereavement, stress in last 2 years |
| n_6145_1_5 | Illness, injury, bereavement, stress in last 2 years |
| n_6145_2_0 | Illness, injury, bereavement, stress in last 2 years |
| n_6145_2_1 | Illness, injury, bereavement, stress in last 2 years |
| n_6145_2_2 | Illness, injury, bereavement, stress in last 2 years |
| n_6145_2_3 | Illness, injury, bereavement, stress in last 2 years |
| n_6145_2_4 | Illness, injury, bereavement, stress in last 2 years |
| n_6145_2_5 | Illness, injury, bereavement, stress in last 2 years |
| n_10721_0_0 | Illness, injury, bereavement, stress in last 2 years (pilot) |
| n_10721_0_1 | Illness, injury, bereavement, stress in last 2 years (pilot) |
| n_10721_0_2 | Illness, injury, bereavement, stress in last 2 years (pilot) |
| n_10721_0_3 | Illness, injury, bereavement, stress in last 2 years (pilot) |
| n_10721_0_4 | Illness, injury, bereavement, stress in last 2 years (pilot) |
| n_21021_0_0 | Pulse wave Arterial Stiffness index |
| n_21021_1_0 | Pulse wave Arterial Stiffness index |
| n_21021_2_0 | Pulse wave Arterial Stiffness index |
| n_42001_0_0 | Source of first myocardial infarction report |
| n_42003_0_0 | Source of first STEMI report |
| n_42005_0_0 | Source of first NSTEMI report |
| n_42007_0_0 | Source of first stroke report |
| n_42009_0_0 | Source of first ischaemic stroke report |
| n_42011_0_0 | Source of first intracerebral haemorrhage report |
| n_42013_0_0 | Source of first subarachnoid haemorrhage report |
| ts_42000_0_0 | Date of first myocardial infarction |
| ts_42002_0_0 | Date of first STEMI |
| ts_42004_0_0 | Date of first NSTEMI |
| ts_42006_0_0 | Date of first stroke |
| ts_42008_0_0 | Date of first ischaemic stroke |
| ts_42010_0_0 | Date of first intracerebral haemorrhage |
| ts_42012_0_0 | Date of first subarachnoid haemorrhage |
| n_2443_0_0 | Diabetes diagnosed by doctor |
| n_2443_1_0 | Diabetes diagnosed by doctor |
| n_2443_2_0 | Diabetes diagnosed by doctor |
| n_2453_0_0 | Cancer diagnosed by doctor |
| n_2453_1_0 | Cancer diagnosed by doctor |
| n_2453_2_0 | Cancer diagnosed by doctor |
| n_2463_0_0 | Fractured/broken bones in last 5 years |
| n_2463_1_0 | Fractured/broken bones in last 5 years |
| n_2463_2_0 | Fractured/broken bones in last 5 years |
| n_2473_0_0 | Other serious medical condition/disability diagnosed by doctor |
| n_2473_1_0 | Other serious medical condition/disability diagnosed by doctor |
| n_2473_2_0 | Other serious medical condition/disability diagnosed by doctor |
| n_6150_0_0 | Vascular/heart problems diagnosed by doctor |
| n_6150_0_1 | Vascular/heart problems diagnosed by doctor |
| n_6150_0_2 | Vascular/heart problems diagnosed by doctor |
| n_6150_0_3 | Vascular/heart problems diagnosed by doctor |
| n_6150_1_0 | Vascular/heart problems diagnosed by doctor |
| n_6150_1_1 | Vascular/heart problems diagnosed by doctor |
| n_6150_1_2 | Vascular/heart problems diagnosed by doctor |
| n_6150_1_3 | Vascular/heart problems diagnosed by doctor |
| n_6150_2_0 | Vascular/heart problems diagnosed by doctor |
| n_6150_2_1 | Vascular/heart problems diagnosed by doctor |
| n_6150_2_2 | Vascular/heart problems diagnosed by doctor |
| n_6150_2_3 | Vascular/heart problems diagnosed by doctor |
