# Supplementary material for: Cross-sectional and longitudinal associations between active commuting and patterns of movement behaviour during discretionary time: A compositional data analysis
Source: PLoS One. 2019 Aug 16;14(8):e0216650. doi: 10.1371/journal.pone.0216650 (PMC6697339; doi:10.1371/journal.pone.0216650)
Supplement: S1 File — (DOC) [file pone.0216650.s005.doc]

STROBE Statement—checklist of items that should be included in reports of observational studies

|  | Item No | Recommendation | Manuscript  pg no |
| --- | --- | --- | --- |
| **Title and abstract** | 1 | (*a*) Indicate the study’s design with a commonly used term in the title or the abstract | 1-2 |
|  |  | **Relevant text**  Pg 1, title: “Cross-sectional and longitudinal associations between active commuting and patterns of movement behaviour during discretionary time: a compositional data analysis”  Pg 2, abstract: “We applied compositional data analysis… using linear regression models adjusted for covariates.”  Pg 2, abstract: “Methods and Findings: We analysed cross-sectional and longitudinal data …” |  |
|  |  | (*b*) Provide in the abstract an informative and balanced summary of what was done and what was found | 2-3 |
|  |  | **Relevant text**  Pg 2, abstract: “We applied compositional data analysis to test for associations between active commuting and the composition and total amount of discretionary time, using linear regression models adjusted for covariates... Active commuting was associated with a more favourable pattern of movement behaviour during discretionary time. Active commuters accumulated 30-60 minutes less screen time per week than those using inactive modes.” |  |
| Introduction | | |  |
| Background/rationale | 2 | Explain the scientific background and rationale for the investigation being reported | 4-5 |
|  |  | **Relevant text**  Pg 4, Introduction: “Movement behaviours (i.e. physical activity and sedentary behaviour) are associated with health in adults.[1-3]… In the travel domain, active travel, or its constituent active commuting (walking or cycling to work) have been associated with favourable health outcomes,[9-11] whilst car use may pose a risk to cardiometabolic health.[12]. Physical activity and sedentary behaviour within the leisure domain, such as sport[13] and screen time[3] respectively have also been linked to health markers.”  Pg 4, Introduction: “Active living approaches seek to promote physical activity and reduce sedentary time across different domains.[15] However, there is little information on how movement behaviours in different domains relate to each other…For example, evidence suggests that increases in active commuting are not compensated for by reductions in leisure physical activity,[16,17] and that active travel is associated with relatively higher leisure physical activity and lower screen time.[18] This preliminary research indicates that active travel is associated with a broadly health-promoting pattern of behaviour overall.” |  |
| Objectives | 3 | State specific objectives, including any prespecified hypotheses | 5 |
|  |  | **Relevant text**  Pg 5, Introduction: “Using a large epidemiological cohort, the aims of this compositional data analysis were to explore:  The cross-sectional relationship between active commuting and the relative composition of discretionary time, incorporating recreational walking, sport and do-it-yourself (DIY) activities, and screen time  The cross-sectional relationship between active commuting and the absolute amount of discretionary time  The longitudinal relationship between changes in active commuting and the relative composition, and absolute amount, of discretionary time.” |  |
| Methods | | |  |
| Study design | 4 | Present key elements of study design early in the paper | 6, 13 |
|  |  | **Relevant text**  Pg 6, Methods: “UK Biobank is a large prospective cohort of British adults aged 40-69 years...”  Pg 13, Methods: “The analysis consisted of three stages. First, we tested for an association between commute mode (inactive vs active) and one of the sets of ilr coordinates representing the discretionary time subcomposition. We used linear regression models (MANCOVA) progressively adjusted for the covariates described above. ..Second, we tested for an association between commute mode (inactive vs active) and the first coordinate of each of the three sets of ilr coordinates (Figure 1) using adjusted linear regression models. This gave an indication of whether screen time, walking for pleasure, or sport/DIY differed between those who used active travel modes and those who used inactive travel modes, relative to the other parts. Third, we tested for an association between commute mode (inactive vs active) and total discretionary time (log transformed because of skew and to satisfy the assumption that the variable can take both positive and negative values), using adjusted linear regression models. This gave an indication of whether total discretionary time differed between those who used active travel modes and those who used inactive travel modes, regardless of the composition of discretionary time.” |  |
| Setting | 5 | Describe the setting, locations, and relevant dates, including periods of recruitment, exposure, follow-up, and data collection | 6 |
|  |  | **Relevant text**  Pg 6, Methods: “UK Biobank is a large prospective cohort of British adults aged 40-69 years. Potential participants in this age group and living in proximity to one of 22 assessment centres across the country were identified from National Health Service registers. The response rate was 5.5%, with 502,633 participants attending a baseline assessment visit between March 2006 and October 2010, which included completion of an electronic touch screen questionnaire.[20] All participants provided written informed consent. More details on the design and methods of the baseline assessment can be found elsewhere.[21,22] The first repeat assessment was carried out between December 2009 and June 2013 (n=20,346) and the second between April 2014 and November 2016 (n=11,923).[23] All repeat assessments included repeat administration of the electronic questionnaire and were restricted only to participants who lived near a single assessment centre in the north of England, Stockport.” |  |
| Participants | 6 | (*a*) *Cohort study*—Give the eligibility criteria, and the sources and methods of selection of participants. Describe methods of follow-up  *Case-control study*—Give the eligibility criteria, and the sources and methods of case ascertainment and control selection. Give the rationale for the choice of cases and controls  *Cross-sectional study*—Give the eligibility criteria, and the sources and methods of selection of participants | 6 |
|  |  | **Relevant text**  Pg 6, Methods: “Potential participants in this age group and living in proximity to one of 22 assessment centres across the country were identified from National Health Service registers… All repeat assessments included repeat administration of the electronic questionnaire and were restricted only to participants who lived near a single assessment centre in the north of England, Stockport.” |  |
|  |  | (*b*) *Cohort study*—For matched studies, give matching criteria and number of exposed and unexposed  *Case-control study*—For matched studies, give matching criteria and the number of controls per case |  |
| Variables | 7 | Clearly define all outcomes, exposures, predictors, potential confounders, and effect modifiers. Give diagnostic criteria, if applicable | 6-7, 12 |
|  |  | **Relevant text**  Pg 6, Methods: “At all assessments, participants who reported being self-employed or in paid employment answered questions about their mode of travel to work… commute mode was dichotomised as inactive (car only) or active (any other mode or combination of modes)… For the longitudinal analysis four categories were created: (i) car only at both observations (stable inactive); (ii) use of any other mode than car at both observations (stable active); (iii) switch from car only to any other mode (inactive to active); or (iv) switch from any other mode to car only (active to inactive).[24,25]”  Pg 7, Methods: “At all assessments, participants answered questions about their sedentary behaviour and physical activity during discretionary time… Participants reported whether they undertook five activities during the preceding four weeks: (i) walking for pleasure (not as a means of travel); (ii) strenuous sports (described as activities that make you sweat or breathe hard); (iii) other less strenuous activities such as swimming or fitness classes; (iv) light do-it-yourself (DIY) activities such as watering the lawn; and (v) heavy DIY activities such as chopping wood or lifting heavy objects.”  Pg 12, Methods: “The covariates were: weekly frequency of travel, the distance in miles between home and work, age, sex, ethnicity, home ownership, car ownership, income, education level, children in the household, Townsend score (an indicator of material deprivation calculated according to home postcode), body mass index, whether job entailed standing, walking or manual labour, bone fracture in the last five years, ever being diagnosed with a vascular condition such as a heart attack or stroke, and ever being diagnosed with a non-vascular condition such as diabetes or cancer.” |  |
| Data sources/ measurement | 8* | For each variable of interest, give sources of data and details of methods of assessment (measurement). Describe comparability of assessment methods if there is more than one group | 6-7 |
|  |  | **Relevant text**  Pg 6, Methods: “At all assessments, participants who reported being self-employed or in paid employment answered questions about their mode of travel to work, with four response options: (i) car or motor vehicle; (ii) public transport; (iii) walk and (iv) cycle. Participants could select a single mode or a combination of modes. Participants also reported the weekly frequency of travel, and the distance (miles), between home and work. Adults who commuted less than once a week or for zero miles were assumed to work from home and were excluded from analysis. Those who reported not being able to walk for any reason were also excluded.”  Pg 7, Methods: “At all assessments, participants answered questions about their sedentary behaviour and physical activity during discretionary time. Participants reported how many hours they spent watching television and using a computer outside of work on a typical day (open-ended question, not distinguishing between week and weekend days). These were summed to produce daily screen time and truncated at nine hours per day.[14] Estimates were converted to minutes per week. Participants reported whether they undertook five activities during the preceding four weeks: (i) walking for pleasure (not as a means of travel); (ii) strenuous sports (described as activities that make you sweat or breathe hard); (iii) other less strenuous activities such as swimming or fitness classes; (iv) light do-it-yourself (DIY) activities such as watering the lawn; and (v) heavy DIY activities such as chopping wood or lifting heavy objects. Where participants reported having undertaken any activity, they reported the frequency and duration according to pre-specified categories (e.g. ‘2-3 times a week’ for ‘15-30 minutes’). Monthly frequencies were divided to equate to weekly frequencies and durations coded according to the mid-point of the category (e.g. 22.5 minutes for those who responded ‘15-30 minutes’). Frequency was multiplied by duration to give minutes per week in each of the five activities.” |  |
| Bias | 9 | Describe any efforts to address potential sources of bias | 13 |
|  |  | **Relevant text**  Pg 13, Methods: “We used linear regression models (MANCOVA) progressively adjusted for the covariates described above.” |  |
| Study size | 10 | Explain how the study size was arrived at | 6 |
|  |  | **Relevant text**  Pg 6, Methods: “The response rate was 5.5%, with 502,633 participants attending a baseline assessment visit between March 2006 and October 2010.” |  |
| Quantitative variables | 11 | Explain how quantitative variables were handled in the analyses. If applicable, describe which groupings were chosen and why | 13 |
|  |  | **Relevant text**  Pg 13, Methods: “We used linear regression models (MANCOVA) progressively adjusted for the covariates described above.” |  |
| Statistical methods | 12 | (*a*) Describe all statistical methods, including those used to control for confounding | 13-14 |
|  |  | **Relevant text**  Pg 13, Methods: “The analysis consisted of three stages. First, we tested for an association between commute mode (inactive vs active) and one of the sets of ilr coordinates representing the discretionary time subcomposition. We used linear regression models (MANCOVA) progressively adjusted for the covariates described above. The MANCOVA findings would be identical using any of the three sets of ilr coordinates. This analysis gave an indication of whether the discretionary time subcomposition differed overall between those who used active travel modes and those who used inactive travel modes. However, it did not identify which particular parts (i.e. screen time, walking for pleasure, or sport/DIY) were driving these differences.  Second, we tested for an association between commute mode (inactive vs active) and the first coordinate of each of the three sets of ilr coordinates (Figure 1) using adjusted linear regression models. This gave an indication of whether screen time, walking for pleasure, or sport/DIY differed between those who used active travel modes and those who used inactive travel modes, relative to the other parts.  Third, we tested for an association between commute mode (inactive vs active) and total discretionary time (log transformed because of skew and to satisfy the assumption that the variable can take both positive and negative values), using adjusted linear regression models. This gave an indication of whether total discretionary time differed between those who used active travel modes and those who used inactive travel modes, regardless of the composition of discretionary time.  Finally, we used the models to predict adjusted compositional means for those who used active travel modes and those who used inactive travel modes. Using the R package lsmeans,[35] we estimated the adjusted mean ilr coordinate value for each of the two ilr coordinates comprising a set. Subsequently, we back-transformed the ilr set using the same ilr partitioning system, firstly into proportions. We then transformed the proportions into minutes per week based on the adjusted mean value of total discretionary time in those who used active travel modes and those who used inactive travel modes separately.”  Pg 14, Methods: “[For the longitudinal analysis] We followed the same general approach described for the cross-sectional analysis. We conducted an initial descriptive analysis of all variables. We then tested for associations between change in commute mode (stable inactive, stable active, inactive to active, active to inactive) and (i) change in the overall discretionary time subcomposition; (ii) relative changes in screen time, walking for pleasure, and sport/DIY; and (iii) change in total discretionary time. We used linear regression models progressively adjusted for the baseline value of the covariates described. Additionally, we derived a continuous variable of the time elapsed between baseline and first repeat assessment, defined according to the dates of assessment; and a variable indicating whether the season differed between assessments. We used these two variables as additional covariates in the maximally adjusted models. For all outcomes, we used the follow-up value adjusted for the baseline value to represent change over time. Finally, we used the models to predict adjusted compositional means for the different commute categories.” |  |
|  |  | (*b*) Describe any methods used to examine subgroups and interactions | N/A |
|  |  | (*c*) Explain how missing data were addressed | 12, 14, 15 |
|  |  | **Relevant text**  Pg 12, Methods: “Cross-sectional analysis: We used baseline data from all participants who provided complete information on exposure (active commuting) and outcomes (composition and total amount of discretionary time), as well as complete information on all covariates.”  Pg 14, Methods: “Longitudinal analysis: We used data from all participants who provided complete information on exposure, outcomes and covariates at baseline and first repeat assessment.”  Pg 15, Methods: “Sensitivity analyses: Because of the large amount of missing data on income and number of children in the household, we repeated the cross-sectional and longitudinal analyses removing these covariates, which markedly increased the sample size.” |  |
|  |  | (*d*) *Cohort study*—If applicable, explain how loss to follow-up was addressed  *Case-control study*—If applicable, explain how matching of cases and controls was addressed  *Cross-sectional study*—If applicable, describe analytical methods taking account of sampling strategy | 7 |
|  |  | **Relevant text**  Pg 7, Methods: “All repeat assessments included repeat administration of the electronic questionnaire and were restricted only to participants who lived near a single assessment centre in the north of England, Stockport.” |  |
|  |  | (*e*) Describe any sensitivity analyses | 15 |
|  |  | **Relevant text**  Pg 15, Methods: “Sensitivity analyses: Because of the large amount of missing data on income and number of children in the household, we repeated the cross-sectional and longitudinal analyses removing these covariates, which markedly increased the sample size.” |  |

| Results | | | Manuscript  pg no |
| --- | --- | --- | --- |
| Participants | 13* | (a) Report numbers of individuals at each stage of study—eg numbers potentially eligible, examined for eligibility, confirmed eligible, included in the study, completing follow-up, and analysed | 6, 16 |
|  |  | **Relevant text**  Pg 6, Methods: “The response rate was 5.5%, with 502,633 participants attending a baseline assessment visit  Pg 16, Results: “From an initial sample of 502,617 participants who provided baseline data, we firstly limited the sample to 246,110 participants who were employed, worked outside the home, reported a commute mode and were able to walk. Following that, we limited the sample to 243,954 participants who had reported some discretionary and screen time, but not more than 24 hours per day of discretionary time. We then limited the sample to those providing complete information on all covariates, leaving a final cross-sectional sample of 182,406 participants.  From an initial sample of 20,346 participants who provided information at both baseline and first repeat assessment, the longitudinal sample was firstly limited to 6,201 participants who provided complete information on exposures, then to 6,133 participants who provided complete information on outcomes, with the final sample limited to 4,323 participants who additionally provided complete information on covariates. The baseline characteristics of the cross-sectional and longitudinal samples are shown in Table 1.  For the sensitivity analysis, the sample size was 237,036 for the cross-sectional analysis and 5,967 for the longitudinal analysis. |  |
|  |  | (b) Give reasons for non-participation at each stage | N/A |
|  |  | (c) Consider use of a flow diagram | N/A |
| Descriptive data | 14* | (a) Give characteristics of study participants (eg demographic, clinical, social) and information on exposures and potential confounders | 16-17, 19, 22 |
|  |  | **Relevant text**  Pg 16, Results: “The baseline characteristics of the cross-sectional and longitudinal samples are shown in Table 1.”  Pg 17: Table 1  Pg 19, Results: “The cross-sectional sample were predominantly middle-aged, White and high socioeconomic status across a range of factors (education, income, home ownership and car ownership). Two thirds (65%) commuted by inactive modes (car only) at baseline. The cross-sectional sample differed from the rest of the baseline sample in all demographic and health characteristics, consistent with an employed population that was the focus of this analysis (Supplementary table 1).”  Pg 22, Results: “The longitudinal sample differed significantly from the rest of the cross-sectional sample in all of the variables listed in Table 1 apart from sex, children in the household and weekly frequency of commuting. Again, the longitudinal sample were predominantly middle-aged, White and high socioeconomic status, and even more so than the cross-sectional sample. Nearly three quarters (72%) commuted by inactive modes (car only) at baseline. Of the 4,323 participants included in analysis, 2,783 (64%) were stable inactive (i.e. car only at both observations), 902 (21%) were stable active (i.e. use of any other mode than car at both observations), 348 (8%) switched from inactive to active modes, and 290 (7%) switched from active to inactive modes. On average, the time elapsed between assessments was 4.3 (standard deviation [SD] 0.9) years. 73% of participants completed the follow-up assessment in a different season to the baseline assessment.” |  |
|  |  | (b) Indicate number of participants with missing data for each variable of interest | 16 |
|  |  | **Relevant text**  Pg 16, Results: “From an initial sample of 502,617 participants who provided baseline data, we firstly limited the sample to 246,110 participants who were employed, worked outside the home, reported a commute mode and were able to walk. Following that, we limited the sample to 243,954 participants who had reported some discretionary and screen time, but not more than 24 hours per day of discretionary time. We then limited the sample to those providing complete information on all covariates, leaving a final cross-sectional sample of 182,406 participants.  From an initial sample of 20,346 participants who provided information at both baseline and first repeat assessment, the longitudinal sample was firstly limited to 6,201 participants who provided complete information on exposures, then to 6,133 participants who provided complete information on outcomes, with the final sample limited to 4,323 participants who additionally provided complete information on covariates. The baseline characteristics of the cross-sectional and longitudinal samples are shown in Table 1.  For the sensitivity analysis, the sample size was 237,036 for the cross-sectional analysis and 5,967 for the longitudinal analysis.” |  |
|  |  | (c) *Cohort study*—Summarise follow-up time (eg, average and total amount) | 22 |
|  |  | **Relevant text**  Pg 22, Results: “On average, the time elapsed between assessments was 4.3 (standard deviation [SD] 0.9) years.” |  |
| Outcome data | 15* | *Cohort study*—Report numbers of outcome events or summary measures over time |  |
|  |  | *Case-control study—*Report numbers in each exposure category, or summary measures of exposure |  |
|  |  | *Cross-sectional study—*Report numbers of outcome events or summary measures | 19-20, 22-23 |
|  |  | **Relevant text**  Pg 19, Results: “Screen time comprised the vast majority (95%) of discretionary time, with a compositional mean of 1635 minutes per week or approximately four hours per day.”  Pg 20: Table 2  Pg 22, Results: “Screen time and walking for pleasure increased over time, whereas sport and DIY activities decreased.”  Pg 23: Table 5 |  |
| Main results | 16 | (*a*) Give unadjusted estimates and, if applicable, confounder-adjusted estimates and their precision (eg, 95% confidence interval). Make clear which confounders were adjusted for and why they were included | 20, 23-24 |
|  |  | **Relevant text**  Pg 20: Table 3  Pg 20: Table 4  Pg 23: Table 6  Pg 24: Table 7 |  |
|  |  | (*b*) Report category boundaries when continuous variables were categorized | N/A |
|  |  | (*c*) If relevant, consider translating estimates of relative risk into absolute risk for a meaningful time period | N/A |
| Other analyses | 17 | Report other analyses done—eg analyses of subgroups and interactions, and sensitivity analyses | 26 |
|  |  | **Relevant text**  Pg 26, Results: “Sensitivity analyses: The cross-sectional and longitudinal sensitivity analyses indicated the same pattern of findings as the main analysis (Supplementary tables 2 & 3), with the exception that the finding on change in total discretionary time remained statistically significant in the maximally adjusted model in the longitudinal sensitivity analysis.” |  |
| Discussion | | |  |
| Key results | 18 | Summarise key results with reference to study objectives | 27 |
|  |  | **Relevant text**  Pg 27, Discussion: “Overall, active commuting was associated with patterns of movement behaviour during discretionary time that appear favourable to health… The largest differences were found for screen time; those using active modes engaged in 30-60 minutes less screen time per week than those using inactive modes. Though modest, this represents a relative energy deficit of approximately 22.5 metabolic equivalent (MET) minutes per week or 0.15% of total daily energy expenditure, and could have a cumulative effect on health over time.” |  |
| Limitations | 19 | Discuss limitations of the study, taking into account sources of potential bias or imprecision. Discuss both direction and magnitude of any potential bias | 28 |
|  |  | **Relevant text**  Pg 28, Discussion: “We also acknowledge the study limitations. UK Biobank is not representative of the UK general population, with evidence of a ‘healthy volunteer’ selection bias,[37] and the longitudinal sample was drawn from only one geographical area. Using the self-reported data available, we were not able to account for all daily activities and therefore construct a complete composition; instead, we focussed on a subcomposition of specific activities occurring during discretionary time. Self-report is subject to recall and social desirability biases, but was necessary in order to explore behaviour at the domain level. The questions used to capture activities in this study had different reference time frames (a typical day for screen time, and the preceding four weeks for the other activities), although it is unlikely this would have changed the results.[38] During data cleaning, we were required to make estimations and decisions about truncating values, though we have been transparent about these decisions and the basis for them. Finally, as exposures and outcomes, and changes in them, were measured concurrently, reverse causation is possible.” |  |
| Interpretation | 20 | Give a cautious overall interpretation of results considering objectives, limitations, multiplicity of analyses, results from similar studies, and other relevant evidence | 29 |
|  |  | **Relevant text**  Pg 29, Discussion: “In conclusion, we found that active commuting was associated with relatively less screen-based sedentary behaviour during discretionary time.” |  |
| Generalisability | 21 | Discuss the generalisability (external validity) of the study results | 28 |
|  |  | **Relevant text**  Pg 28, Discussion: “UK Biobank is not representative of the UK general population, with evidence of a ‘healthy volunteer’ selection bias,[37] and the longitudinal sample was drawn from only one geographical area.” |  |
| Other information | | |  |
| Funding | 22 | Give the source of funding and the role of the funders for the present study and, if applicable, for the original study on which the present article is based | 30 |
|  |  | **Relevant text**  Pg 30, Acknowledgments: “This research was conducted using the UK Biobank resource (application No 20684). LF was funded by the Centre for Diet and Activity Research (CEDAR), a UKCRC Public Health Research Centre of Excellence. Funding from the British Heart Foundation, Cancer Research UK, Economic and Social Research Council, Medical Research Council, the National Institute for Health Research, and the Wellcome Trust, under the auspices of the UK Clinical Research Collaboration, is gratefully acknowledged (087636/Z/08/Z, ES/G007462/1, MR/K023187/1). DO (MC_UU_12015/6) and KW (MC_UU_12015/3) were supported by the Medical Research Council. We acknowledge Craig Knott for his assistance with data cleaning and variable derivation. The funders played no role in the study design; in the collection, analysis, and interpretation of data; in the writing of the paper; or in the decision to submit the paper for publication.” |  |

*Give information separately for cases and controls in case-control studies and, if applicable, for exposed and unexposed groups in cohort and cross-sectional studies.

**Note:** An Explanation and Elaboration article discusses each checklist item and gives methodological background and published examples of transparent reporting. The STROBE checklist is best used in conjunction with this article (freely available on the Web sites of PLoS Medicine at http://www.plosmedicine.org/, Annals of Internal Medicine at http://www.annals.org/, and Epidemiology at http://www.epidem.com/). Information on the STROBE Initiative is available at www.strobe-statement.org.
